# Hybridity has a greater effect than paternal genome dosage on heterosis in sugar beet (*Beta vulgaris*)

**DOI:** 10.1186/s12870-018-1338-x

**Published:** 2018-06-15

**Authors:** Brendan F. Hallahan, Eva Fernandez-Tendero, Antoine Fort, Peter Ryder, Gilles Dupouy, Marc Deletre, Edna Curley, Galina Brychkova, Britta Schulz, Charles Spillane

**Affiliations:** 1 Genetics and Biotechnology Laboratory, Plant and AgriBioscience Research Centre (PABC), Ryan Institute, National University of Ireland Galway, University Road, Galway, H91 REW4 Ireland; 2 Sugar Beet Genomics, KWS SAAT AG, Einbeck, Germany

**Keywords:** Hybrid vigor, Heterosis, Hybridity, Polyploidy, Genome dosage, Triploid, F1 hybrid

## Abstract

**Background:**

The phenomenon of heterosis is critical to plant breeding and agricultural productivity. Heterosis occurs when F1 hybrid offspring display quantitative improvements in traits to levels that do not occur in the parents. Increasing the genome dosage (i.e. ploidy level) of F1 offspring can contribute to heterosis effects. Sugar beet (*Beta vulgaris*) provides a model for investigating the relative effects of genetic hybridity and genome dosage on heterosis. Sugar beet lines of different ploidy levels were crossed to generate diploid and triploid F1 offspring to investigate the effect of; (1) paternal genome dosage increase on F1 heterosis, and; (2) homozygous versus heterozygous tetraploid male parents on F1 triploid heterosis. A range of traits of agronomic and commercial importance were analyzed for the extent of heterosis effects observed in the F1 offspring.

**Results:**

Comparisons of parental lines to diploid (EA, EB) and triploid (EAA, EBB) F1 hybrids for total yield, root yield, and sugar yield indicated that there was no effect of paternal genome dosage increases on heterosis levels, indicating that hybridity is the main contributor to the heterosis levels observed. For all traits measured (apart from seed viability), F1 triploid hybrids derived from heterozygous tetraploid male parents displayed equivalent levels of heterosis as F1 triploid hybrids generated with homozygous tetraploid male parents, suggesting that heterosis gains in F1 triploids do not arise by simply increasing the extent of multi-locus heterozygosity in sugar beet F1 offspring.

**Conclusions:**

Overall, our study indicates that; (1) increasing the paternal genome dosage does not enhance heterosis in F1 hybrids, and; (2) increasing multi-locus heterozygosity using highly heterozygous paternal genomes to generate F1 triploid hybrids does not enhance heterosis. Our findings have implications for the design of future F1 hybrid improvement programs for sugar beet.

**Electronic supplementary material:**

The online version of this article (10.1186/s12870-018-1338-x) contains supplementary material, which is available to authorized users.

## Background

Heterosis can be described as an increase in size or other desirable characteristics (e.g. grain yield) in the F1 offspring beyond that observed in the parental lines [1]. The application of heterosis for the improvement of specific traits in crops is highlighted by the widespread development of F1 hybrid varieties and their widespread adoption by farmers [1]. Most crop plants are polyploids, either autopolyploids or allopolyploids [2]. Polyploidy (i.e. genome dosage changes) can also be harnessed for crop improvement, as some polyploids can display fitness advantages over progenitor or parental lines. During crop domestication, and subsequent artificial selection, polyploids may have been selected for due to desirable traits such as gigantism [3]. Genome dosage increases in newly formed polyploids can elicit novel phenotypes, while genetic redundancy within polyploid genomes can allow for neo- or subfunctionalization of gene functions [4, 5].

Heterosis effects can arise as a result of both gene and genome dosage effects [6–9], particularly in allopolyploid plants (which are also by definition hybrids) [7, 10]. The disentanglement of parental genome dosage versus hybridity contributions to heterosis requires the use of genome dosage series lines (genotypes) which are ideally genetically identical F1 hybrids, yet contain additional chromosome sets from either one or the other parent [11–14]. Such experiments have been done in *Arabidopsis thaliana* [11, 15–17] and in maize (*Zea mays*) [18, 19] revealing significant parental genome dosage effects on heterosis.

Parental lines which elicit significant heterosis effects are identified empirically through crossing experiments to identify parental germplasm pools that display good combining ability to generate heterosis effects in F1 hybrids [1]. While it could be considered that the genetic distance between parental lines could be used as basis to select parental lines to generate superior heterozygous F1 hybrids, there is rather limited evidence to support this approach. Early investigations into parental genetic distance and F1 hybrid performance in maize found a general correlation between genetic distance and heterosis up to a certain threshold. By grouping parental germplasm based on regional adaptation, F1 heterosis increased with increased parental divergence within a range: parents from different parts of the USA when crossed together produced F1 hybrids with considerable heterosis, but when parents from the USA were crossed with Mexican varieties, which represents a wider cross, there was less heterosis [20]. More recent experiments utilizing molecular markers have found no correlation between parental genetic distance and heterosis in maize [21–23]. Other crops where this has been investigated (e.g., bread wheat, rice, pepper, oilseed rape) and in models such as *Arabidopsis thaliana*, have also shown limited or no evidence supporting this approach to selecting parental lines for triggering heterosis effects in F1 hybrids [24–31].

Sugar beet is a crop which is amenable to heterosis comparisons between diploid and triploid genotypes, including whether heterosis effects can be augmented by changing paternal genome dosage or multi-locus heterozygosity in the F1 hybrid. Indeed, commercial sugar beet breeding first began exploiting genome dosage effects in the 1930s [32], where early triploid varieties displayed many favorable agronomic traits, including high yield [33]. Following the advent of cytoplasmic male sterile lines and the introduction of the monogerm seed character (i.e. fruits which produce a single seed) [34], more efficient and reliable F1 hybrid sugar beet production became possible. In North America, sugar beet breeding has largely focused on diploid F1 hybrid varieties whereas in Europe triploid F1 hybrids are historically more popular. However, in recent years European sugar beet breeding programs have increasingly moved toward diploid F1 hybrid breeding [32, 35]. Sugar beet provides a useful model for investigating the contributions of paternal genome dosage versus hybridity to heterosis in a commercial crop.

In this study, we consider three mechanisms that could potentially contribute to sugar beet heterosis, namely ploidy (genome dosage), hybridity and increasing multi-locus nuclear heterozygosity. While clearly interlinked, hybridity and heterozygosity are not synonymous. An F1 hybrid offspring plant is a genetic composite of the maternal cytoplasmic genome and the biparental nuclear genomes inherited from each parents [36]. Heterozygosity is a measure of nuclear genetic diversity, which can be determined by the extent of multi-locus single nucleotide polymorphisms (SNPs) across the nuclear genome [37]. We crossed sugar beet lines of different ploidy levels to generate F1 hybrids allowing investigation of: (1) the effect of paternal genome dosage increase on F1 heterosis, and; (2) the effect of homozygous versus heterozygous tetraploid male parents on F1 hybrid triploid heterosis. Our findings indicate that (1) agronomically important traits such as total yield, root yield and sugar yield are more influenced by hybridity than paternal genome dosage increases, and (2) F1 triploid hybrids with greater levels of multi-locus nuclear heterozygosity do not display improvements for total yield, root yield, and sugar yield. We consider that our findings have relevance to the design of future hybrid breeding programs for sugar beet improvement.

## Methods

### Sugar beet lines and crossing design

Sugar beet lines/genotypes obtained from KWS SAAT consisted of: (a) isogenic and hybrid diploid parental lines; (b) isogenic and hybrid tetraploid parental lines; and (c) F1 progeny (diploid and triploid) offspring generated from the parental lines (Table 1 and Table 2). Double haploids were generated by ovule isolation from an F1 seed, followed by colchicine treatment. Homozygous tetraploids were generated when the double haploids underwent spontaneous doubling during colchicine treatment. The heterozygous tetraploids were selected by KWS SAAT for good performance over many years within the KWS SAAT sugar beet breeding program, where these tetraploids (C^1^C^2^C^3^C^4^ and D^1^D^2^D^3^D^4^) harbor high levels of heterozygosity at each locus across the nuclear genome, hereafter referred to as (CCCC) and (DDDD). All three diploid tester lines (Tester 1, 2, and 3) of sugar beet that were used as female parents were sourced from the monogerm seed parent pool, i.e. two monogerm lines were crossed and the F1 hybrid was back-crossed several times with a cytoplasmic male sterile (CMS) line. The F1 hybrids were created using a female tester and pollinator line in the same plot at KWS SAAT. The F1 hybrid seed was harvested from the male sterile female tester lines.

**Table 1:** Parental sugar beet germplasm

	Genotype	Hybridity status	Experiment ID
2×	Double Haploid (AA)	Homozygous	2× DH (AA)
−	Double Haploid (BB)	Homozygous	2× DH (BB)
	CMS line ‘Tester 1’ (EE)	Heterozygous	2× hybrid (‘Tester 1’) (EE)
	CMS line ‘Tester 2’ (FF)	Heterozygous	2× hybrid (‘Tester 2’) (FF)
	CMS line ‘Tester 3’ (GG)	Heterozygous	2× hybrid (‘Tester 2’) (GG)
4×	Tetraploid (AAAA)	Homozygous	4× (AAAA)
−	Tetraploid (BBBB)	Homozygous	4× (BBBB)
	Tetraploid (CCCC)	Heterozygous	4× hybrid (CCCC)
	Tetraploid (DDDD)	Heterozygous	4× hybrid (DDDD)

*CMS* cytoplasmic male sterile, *DH* double haploid

**Table 2:** Sugar beet parental lines used to generate F1 diploid and F1 triploid hybrid offspring

♂	2× DH (AA)	4× (AAAA)	2× DH (BB)	4× (BBBB)	4× hybrid (CCCC)	4× hybrid (DDDD)
♀						
2× hybrid (‘Tester 1’) (EE)	F1 2× hybrid (EA)	F1 3× hybrid (EAA)	F1 2× hybrid (EB)	F1 3× hybrid (EBB)	−	
2× hybrid (‘Tester 2’) (FF)	−				F1 3× hybrid (FCC)	−
2× hybrid (‘Tester 3’) (GG)	−					F1 3× hybrid (GDD)

*DH* Double Haploid. Different genotypes specified in parentheses. *A* A genotype; *B* B genotype; *C* C genotype; *D* D genotype; *E* genotype E; *F* genotype F; *G* genotype G

### Sugar beet germination and seed viability test

Sugar beet fruits can contain one seed (monogerm) or more than one seed (multigerm). Sugar beet fruits (containing botanical seed) were sown and seed germination was investigated in accordance with International Rules for Seed Testing (ISTA, 2010). Fifty randomly selected fruits of each line were placed is 50 ml distilled water and covered with foil to block any light. These were left for 2 h, rinsed, and placed in Grade 3236 pleated cellulose filtered paper (GE Healthcare, Fairfield, CT, USA) inside a 100 mm × 100 mm × 20 mm petri dish (Sarstedt AG & Co, Nümbrecht, Germany). The paper was cut to size and 6.4 ml of distilled water was added. The petri dishes were sealed with parafilm and placed in a growth chamber (Snijders Scientific, Tilburg, Netherlands) at the beginning of the dark cycle. The growth conditions were 8 h day/16 h night @30 °C/20 °C respectively, in accordance with ISTA guidelines. Germination was visually scored on day 4 and 14 post sowing date. A seed was categorized as ‘germinated’ when the radicle had emerged from the operculum of the fruit. Ungerminated seeds were examined under a Leica MZ microscope (Leica, Wetzlar, Germany) and were categorized as alive if they were plump, while seeds that were wrinkled and poorly formed were classified as dead. Fruits with only dead seeds were excluded from final calculations. The seed germination count of each line was calculated as follows:

$$ \frac{No. of\ fruits\ containing\  at\  least\  one\  germinated\ seed}{No. of\ fruits\ containing\ only\ ungerminated, healthy\ seed s} $$

### Sugar beet seed and fruit analysis

**Fig. 1:**
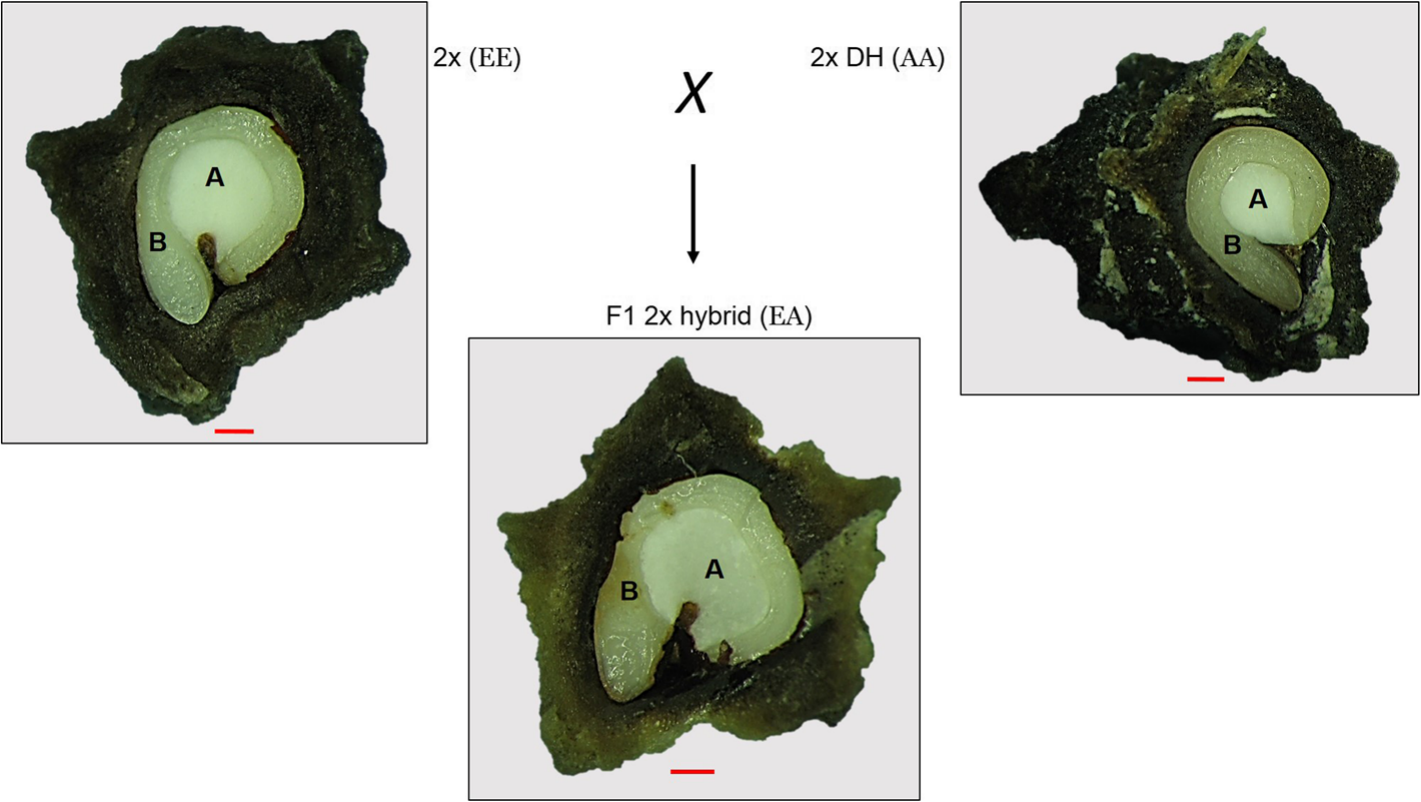
Cross-section of sugar beet fruits revealing seed inside. Representative F1 2× hybrid (EA) and its parent lines. Seed tissue is labelled A perisperm, and, B embryo. Red line is scale of 0.5 mm

For analysis of the seed and empty fruits of sugar beet, 25 randomly selected fruits of each line were placed in 50 ml distilled water for 48 h and covered with foil to block any light. The operculum and seed were removed from the fruit and weighed individually on a weighing scale (Mettler Toledo, Switzerland). Seed size data generated is for alive seeds. The entire experiment was replicated 4 times giving a total of 100 seed measurements for each line. For the analysis of the seed cross-sections, 30 randomly selected fruits of each line were placed in 50 ml distilled water and covered with foil. After 36 h, the operculum and the seed testa (seed coat) were removed with a razor blade to make the embryo and perisperm visible. The cross-section was imaged under a Leica MZ dissecting microscope (Leica, Wetzlar, Germany), and the embryo and perisperm size (i.e. area) were determined using IMAGEJ (US ImageJ, Bethesda, MD, USA) software. See Fig. 1 for a representative example of a cross section of a sugar beet fruit containing a seed.

### Ploidy analysis of sugar beet seedlings

Seedlings of each sugar beet line were grown in individual pots of soil (5:1:1 mixture of peat soil:perlite:vermiculite) and placed in a growth chamber (16 h day/8 h night @21 °C/18 °C). A destructive harvest of first true leaves was performed, where 400 μl of nuclei extraction buffer (Sysmex, Kobe, Japan) was added to the leaf material which was chopped with a razor blade. The chopped mixture was left for 5 min. The mixture was strained through a 30 μm CellTrics® filter (Sysmex, Kobe, Japan) into a 3.5 ml Röhren tube. 40 μl of 1% *v*/v polyvinylpyrrolidone (PVP) was added and left for 5 min. Finally, 1 ml of UV-stain was added to the tube. The sample was analyzed on a Partec Ploidy Analyzer (Sysmex Kobe, Japan). The ploidy level of each line was confirmed.

### DNA extraction

Seedlings of each sugar beet line were grown in individual pots as described previously. A destructive harvest of first true leaves was performed. Genomic DNA (gDNA) was extracted from three biological replicates of each line with a NucleoSpin® Plant II kit (Düren, Germany), according to the manufacturer’s instructions with one revision: incubation time with RNase A was increased to 30 min. The quality and quantity of gDNA was evaluated via agarose gel electrophoresis, NanoDrop® 2000 spectrophotometer (Thermo Fisher Scientific, MA, USA), and Qubit® 2.0 fluorometer (Thermo Fisher Scientific, MA, USA). Sample DNA, with OD_260_/OD_280_ ratio of 1.8 to 2.0 and total amount of more than 1.5 μg, was used for library construction.

### Library preparation, Illumina sequencing and SNP detection for measuring nuclear multi-locus heterozygosity

Circa 0.3~ 0.6 μg of gDNA was digested with restriction enzymes and the resulting digested fragments were ligated to two barcoded adapters, the universal adapter (5’ AATGATACGGCGACCACCGAGATCTACACTCTTTCCCTACACGACGCTCTTCCGATCT 3′) and the indexed adapter (5’ GATCGGAAGAGCACACGTCTGAACTCCAGTCAC-NNNNNN-ATCTCGTATGCCGTCTTCTGCTTG 3′) which when annealed generated compatible sticky ends corresponding to the restriction digestion enzyme. Following PCR amplification, all samples were pooled and size-selected for the required fragments to complete the library construction. Qubit® 2.0 fluorometer was used to determine the DNA concentration of the prepared libraries. After dilution to 1 ng DNA/μl, an Agilent® 2100 bioanalyzer (Agilent Technologies, CA, USA) was used to determine the insert size. Finally, quantitative real-time PCR (qPCR) was performed to determine the effective concentration of each library. If the library with appropriate insert size had an effective concentration of > 2 nM, the constructed libraries were deemed sufficient quality and used for Illumina® high-throughput sequencing.

The DNA libraries were pooled according to their effective concentration as well as the expected data production. Paired-end sequencing was performed on an Illumina®HiSeq platform (Illumina, CA, USA) at Novogene Technology Co., Ltd. (Beijing, China).

Raw sequencing reads were processed with CASAVA software (version 1.8) and sequencing data was assessed for quality distribution, sequencing errors and adapter contamination. In total 29.068Gbit of raw data were sequenced, with 29.065Gbit clean data generated after quality control. The clean sequencing data was aligned with the reference sequence using Burrows-Wheeler Aligner (BWA) software [38] and the mapping rate and coverage was assessed. Sorting of the BAM files was performed with SAMtools software [39]. For the detection and filtration of SNPs and InDels we used SAMtools software [39]. Annotation of detected SNPs was performed with ANNOVAR software [40]. The genome-wide multi-locus heterozygosity rate was calculated by the ratio of heterozygous SNPs to the total number of genome bases. A detailed description of the genotyping-by-sequencing methods is provided in Additional file 1.

### Sugar beet field trial

A field trial of the sugar beet lines was conducted in Cobh, Cork, Ireland (51.851°N 8.2967°W) as a complete randomized block design with 4 replicates. Fruits (seeds) were sown in the first week of April 2015 and harvested in the last week of October 2015. The mean air temperature over the season was 13 °C and mean precipitation was 68.4 mm (Cobh Weather station 51°51’.18 N 008°17’.40 W). Trial plots consisted of 20 rows, each 9 m in length with an inter-row spacing of 61 cm. Each sugar beet line (i.e. genotype) was sowed once per plot. The position of each line was randomized in each plot with a commercial sugar beet variety (cv. Rosalinda from KWS SAAT) occupying the five remaining rows. Three hundred fruits per row were sown manually and thinned out (after homogenous emergence four weeks post sowing date) to a population density of 50 plants per row, thus leaving approximately 18 cm of space between plants.

### Sugar beet harvest procedure

The harvesting of the sugar beet was performed manually. All weight measurements were recorded with a ‘Defender 3000’ weighing scale (Ohaus Corporation, NJ, USA). The total yield (kg/ha) of each line was calculated, plants were left to dry overnight in the field, and thereafter 25 plants from each line were randomly selected and weighed individually. The crown and leaves were both removed by cutting below the lowest leaf scar, and both above- and below-ground parts were weighed separately. Root length was recorded from the top of the root to the root tip and circumference was recorded at the widest part of the root. Twenty-five roots from each line were stored in polypropylene bags and transported under controlled conditions @ 2–7 °C to KWS SAAT SE, Germany for compositional analysis.

### Sugar beet chemical compositional analysis

Raw sugar beet roots were stored according to best practice [41] and analyzed following internationally agreed protocols [42]. Chemical composition was determined with an automatic beet laboratory system (Venema, Groningen, Netherlands). Briefly, roots were washed, weighed and processed into brei. Subsequently, brei was stored at − 26 °C until analysis. The brei sample was clarified with 0.3% *w*/*v* aluminium sulfate solution. Sucrose was measured polarimetrically and Sodium and Potassium by flame photometry. α-amino Nitrogen was analyzed by the fluorometric OPA-method.

### Formulae and statistical analysis

Following chemical analysis, the “new Braunschweig formula” was applied to calculate the loss of sugar to impurities, the resulting sugar content and subsequent sugar yield of each line [43]:

$$ Standard\ Molasses\ Loss\ (SML)=0.12\left( Na+K\right)+0.24\left(\alpha -N\right)+0.48 $$

$$ Corrected\ Sugar\ Content\ (CSC)= SC- SML- SFL $$

$$ Corrected\ Sugar\ yield= beet\ yield\times CSC $$

Where Na + K is the sum of Sodium and Potassium in mmol/100 g of beet, α-N is α-amino Nitrogen in mmol/100 g of beet, SC is sugar content of beet and SFL is Standard Factory Loss of 0.6.

The mid-parent value for all traits was calculated as follows:

$$ \frac{Mean\ of\ Parent\ 1}{\left( Mean\ of\ Parent\ 1\  and\ Parent\ 2\right)} $$

All data points for Parent 1 were normalized around this mid-parent value. A one-tailed independent samples *t*-test was used to determine whether F1 hybrid means were significantly higher or lower than the best-parent or mid-parent mean. A two-way analysis of variance (ANOVA) with a post-hoc Tukey’s HSD test was used to determine the influence of ploidy level and hybridity across different traits in female and male parent lines (Genotypes (EE), (AA), (BB), (AAAA), (BBBB)) and F1 hybrids (Genotypes (EA), (EB), (EAA), (EBB)). A one-way ANOVA with a post-hoc Tukey’s HSD test was used to determine whether the F1 3× hybrids (Genotypes (EAA), (EBB), (FCC), (GDD)) differed for important agronomic traits and for nuclear multi-locus heterozygosity levels.

Additional files 2, 3 and 4 contain Supplementary Results and Full Datasets.

## Results

### F1 diploid hybrids of sugar beet exhibit positive heterosis effects on seed traits

**Fig. 2:**
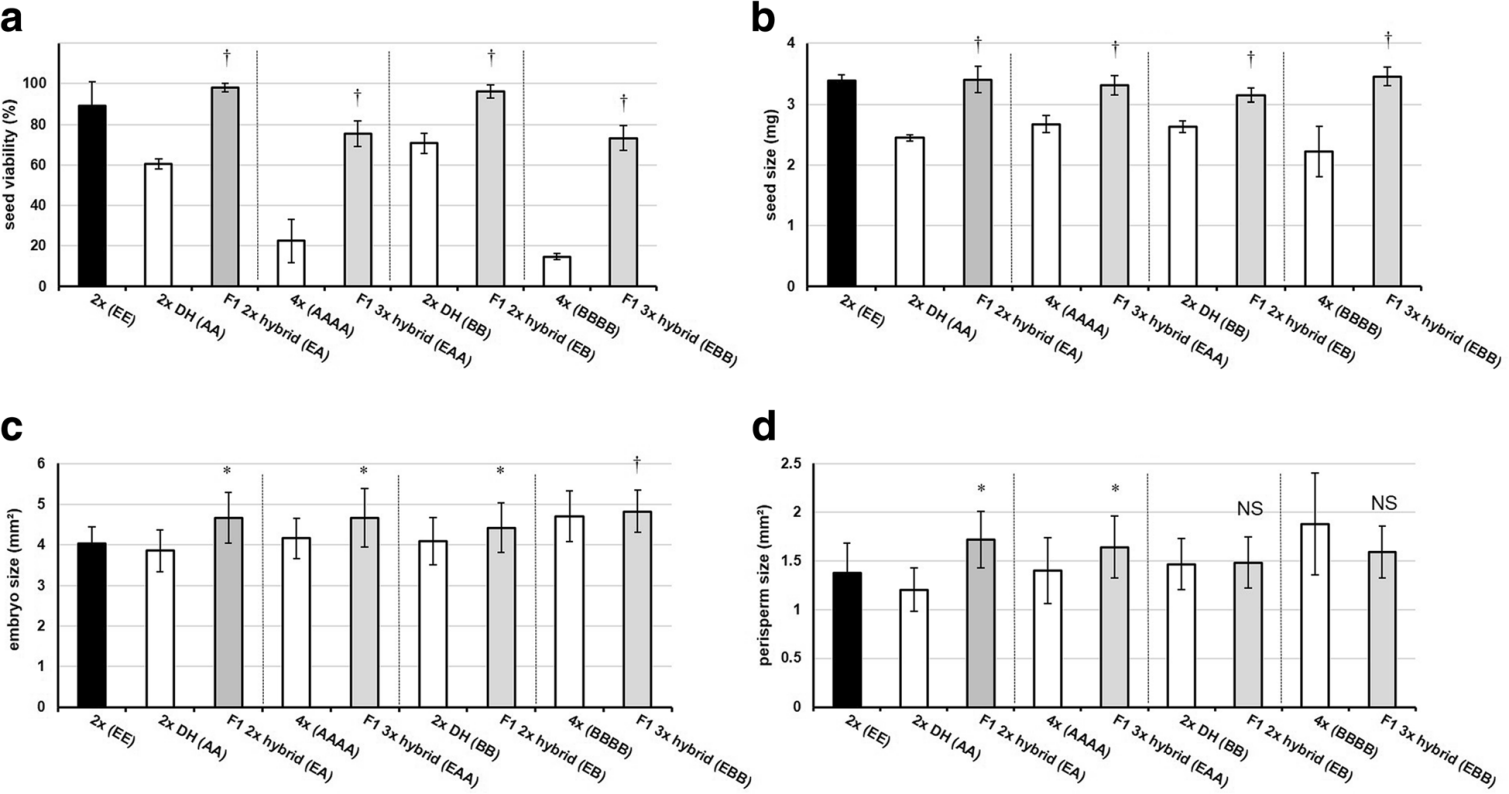
Sugar beet seed characteristics of diploid and triploid F1 hybrids and their parent lines. Data are mean of four replicates (± SD). **a** Seed viability, **b** seed size, **c** embryo size, **d** perisperm size. Different genotypes specified in parentheses. DH = double haploid. * Best parent heterosis (*P* ≤ 0.05), † Mid-parent heterosis (*P* ≤ 0.05), NS Not significantly different (*P* > 0.05)

To determine the extent of heterosis effects on sugar beet seed traits at the diploid level, the viability and size of sugar beet seeds of the parental and F1 generations were analyzed (Table 1 and Table 2). For this analysis sugar beet seeds were removed from their fruits (Fig. 1). The F1 2× hybrids (EA) and (EB) display heterosis effects with respect to seed viability (*P* ≤ 0.05) and seed size (*P* ≤ 0.05) (Fig. 2aand b). To determine the extent of heterosis effects at the diploid level on tissue characteristics of the F1 hybrid seeds, seed cross-sections were investigated. This revealed that the F1 2× hybrid (EA) and (EB) seeds display heterosis (*P* ≤ 0.05) for embryo size (Fig. 2c). While the F1 2× hybrid (EA) seed display heterosis (*P* ≤ 0.05) for perisperm size, there was no significant heterosis effect in perisperm size observed for the F1 2× hybrid (EB) (*P* > 0.05) (Fig. 2d).

### F1 diploid hybrids of sugar beet exhibit positive heterosis for root morphology, yield and sugar yield

**Fig. 3:**
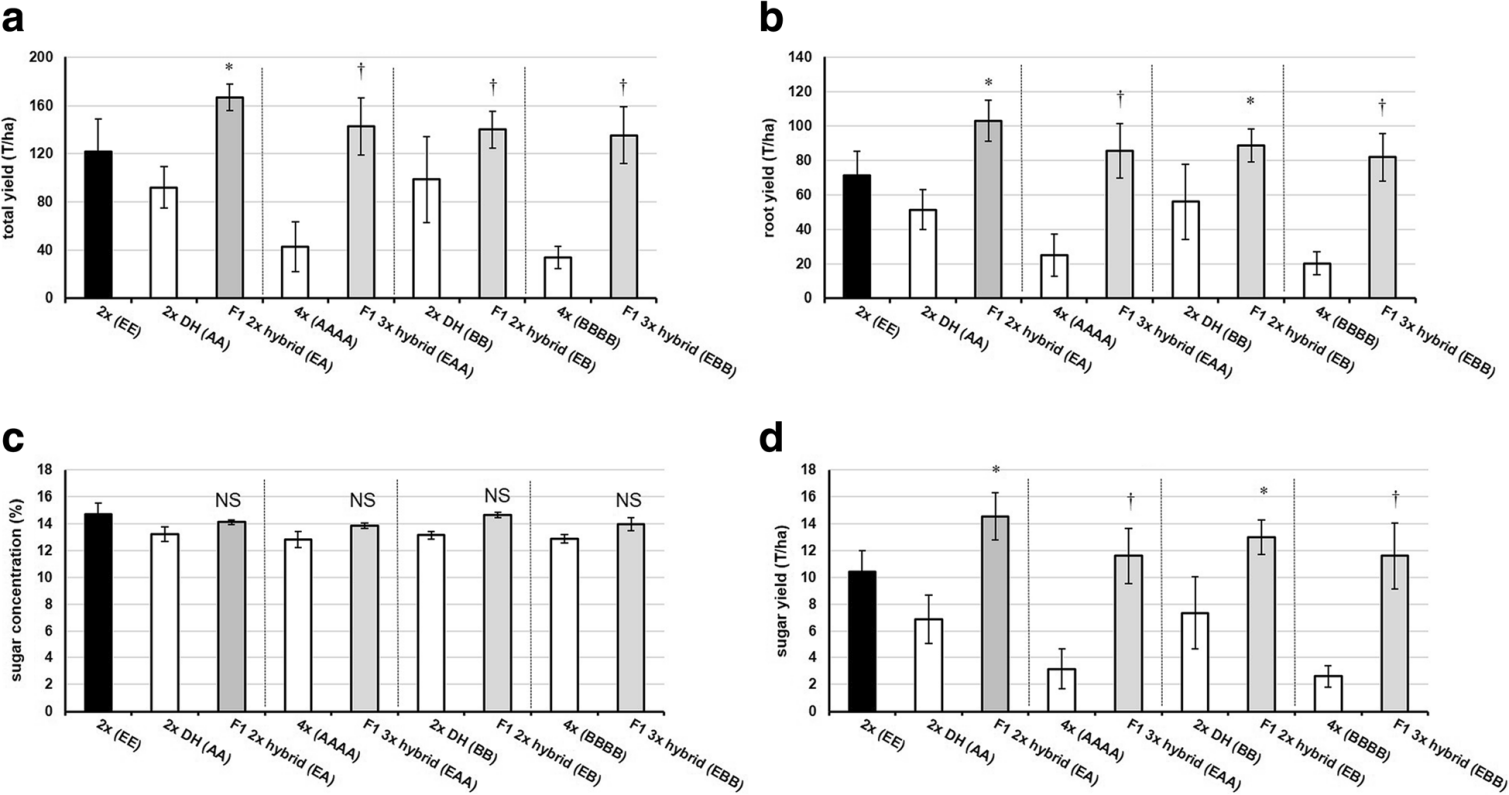
Agronomic and root quality traits of diploid and triploid F1 hybrids of sugar beet and their parental lines. Data are mean of four replicates (± SD). **a** Total yield, **b** root yield, **c** corrected sugar content, **d** corrected sugar yield. Different genotypes specified in parentheses. DH = double haploid. * Best parent heterosis (*P* ≤ 0.05), † Mid-parent heterosis (*P* ≤ 0.05), NS Not significantly different (*P* > 0.05)

To investigate possible heterosis effects on agronomic traits at the diploid level, a field trial of parental and F1 hybrid lines was conducted. At 121 T/ha, the female parent 2× (EE) has the highest yield of the parent lines. Both the F1 2× hybrid (EA) and F1 2× hybrid (EB) display heterosis (*P* ≤ 0.05) for total yield (Fig. 3a). However, both F1 2× hybrids (EA) and (EB) have a harvest plant density that is not significantly different (*P* > 0.05) from their mid-parent values. The F1 2× hybrid (EA) displays heterosis (*P* ≤ 0.05) for above-ground biomass (i.e. the fresh weight of the leaves plus the root crown), whereas the F1 2× hybrid (EB) has an above-ground biomass not significantly different (*P* = 0.15) from the mid-parent value (Additional file 2: Table S2). At 71 T/ha, the female parent 2× (EE) has the highest root yield of the parent lines. Both the F1 2× hybrid (EA) and F1 2× hybrid (EB) display heterosis for root yield (*P* ≤ 0.05) (Fig. 3b). Both of the F1 2× hybrids display heterosis (*P* ≤ 0.05) for root circumference. For root length, only the F1 2× hybrid (EB) shows heterosis (*P* ≤ 0.05) while the F1 2× hybrid (EA) does not have a significantly longer tap root (*P* = 0.10) than the mid-parent value (Additional file 2: Table S2). To determine heterosis effects on root quality traits, the harvested sugar beets were analyzed for chemical composition. The corrected sugar content for all lines ranges between 13 and 14%; the F1 hybrids do not have significantly different sugar content than the mid-parent values (*P* > 0.05) (Fig. 3c). At 10 T/ha, the female parent 2× (EE) has the highest corrected sugar yield of the parent lines. Both the F1 2× hybrid (EA) and F1 2× hybrid (EB) display heterosis for corrected sugar yield (Fig. 3d).

### Paternally-inherited genome dosage increase does not enhance heterosis effects in F1 triploid hybrids relative to F1 diploids hybrids

To determine whether a paternally-inherited genome dosage increase influences heterosis in F1 hybrids, F1 triploids were generated. The sterile mother, genotype (EE), was crossed with a tetraploid pollen parent (AAAA) and (BBBB) to generate F1 triploid offspring. This differed from use of a diploid pollen parent, (AA) and (BB), as used for the F1 diploid hybrids. These F1 triploids, (EAA) and (EBB), are genetically identical (at the DNA sequence level) to the F1 diploids, (EA) and (EB), apart from the triploids having an extra paternally-inherited chromosome set (Table 2).

### F1 triploid hybrids of sugar beet exhibit positive heterosis effects on seed traits

Similar to their equivalent F1 2× hybrids, the F1 3× hybrid (EAA) and (EBB) display heterosis for seed viability (*P* ≤ 0.05) and seed size (*P* ≤ 0.05). The homozygous tetraploid male parents, 4× (AAAA) and (BBBB), have the lowest percentage of alive seeds among the parent lines (23 and 15%, respectively) (Fig. 2a and b). With an embryo size area of 4mm^2^ and 4.7mm^2^ respectively, the homozygous tetraploid male parents 4× (AAAA) and (BBBB) are the best performing parents for F1 embryo size. Both the F1 3× hybrid (EAA) and F1 3× hybrid (EBB) seeds display heterosis (*P* ≤ 0.05) for embryo size (Fig. 2c). The F1 3× hybrid (EAA) exhibits heterosis (*P* ≤ 0.05) for perisperm size, but there is no significant difference between F1 3× hybrid (EBB) and its parent lines for perisperm size (*P* > 0.05). The heterosis effect on perisperm size recorded in F1 triploids mimics that seen in their equivalent F1 diploids (Fig. 2d).

### F1 triploid hybrids of sugar beet exhibit positive heterosis for root morphology, yield and sugar yield

Both F1 3× hybrid (EAA) and F1 3× hybrid (EBB) display heterosis for total yield and root yield (*P* ≤ 0.05) (Fig. 3a and b). Both of the F1 3× hybrids display heterosis (*P* ≤ 0.05) for harvest plant density, likely due to the low field emergence of parents 4× (AAAA) and (BBBB). This contrasts with their equivalent F1 2× hybrids which do not show a greater plant density than their parents. Both F1 3× hybrid (EAA) and F1 3× hybrid (EBB) display heterosis (*P* ≤ 0.05) for above-ground biomass. Like their equivalent F1 2× hybrids, both F1 3× hybrids display heterosis (*P* ≤ 0.05) for root circumference. For root length, both F1 3× hybrid (EAA) and F1 3× hybrid (EBB) display heterosis (*P* ≤ 0.05) (Additional file 2: Table S2). As also seen for both F1 2× hybrids, both F1 3× hybrids do not have significantly different sugar content than their mid-parent values (*P* > 0.05) (Fig. 3c). Both F1 3× hybrids display heterosis (*P* ≤ 0.05) for corrected sugar yield (Fig. 3d).

### F1 hybrids exhibit heterosis for important agronomic traits regardless of ploidy level

Comparisons of the F1 2× and 3× hybrids reveal that there are some differences in the levels of heterosis for certain important agronomic traits. For example, the F1 2× hybrids (EA) and (EB) have close to 100% seed viability whereas the F1 3× hybrid (EAA) and 3× hybrid (EBB) have circa 75% seed viability (Fig. 2a). Also, both F1 2× hybrid (EA) and (EB) show best parent heterosis for root yield and sugar yield while both F1 3× hybrid (EAA) and 3× hybrid (EBB) show only mid-parent heterosis for these traits (Fig. 3b and d).

Using the different levels of heterosis recorded across both F1 2× and 3× hybrids, a two-way analysis of variance (ANOVA) was performed to determine the relative influence of ploidy level and hybridity on important agronomic traits. Both the F1 2× hybrids (EA) and (EB) and both F1 3× hybrids (EAA) and (EBB) and their parent lines (EE), (AA), (BB), (AAAA) and (BBBB) were grouped according to ploidy level (diploid, triploid, tetraploid; factor 1) or hybridity (isogenic, hybrid; factor 2). A Tukey’s HSD test detected any statistically significant difference between ploidy levels. Both ploidy level and hybridity significantly affect all important agronomic traits examined (*P* ≤ 0.05) (Table 3). A Tukey’s HSD test reveals ploidy level has a significant effect on seed viability between diploid and triploid lines (*P* ≤ 0.05), whereas for all other traits examined – total yield, root yield, corrected sugar content, corrected sugar yield – there is no significant effect from ploidy level on diploid and triploid lines (*P* > 0.05) (Table 4). Our data indicates that F1 hybrids perform better than their parents, regardless of their ploidy (genome dosage) level.

**Table 3:** Two-way ANOVA results displaying significant effects of ploidy level and hybridity on important agronomic traits. Both F1 2× hybrids (EA) and (EB) and F1 3× hybrids (EAA) and (EBB) and their parent lines (EE), (AA), (BB), (AAAA) and (BBBB) were grouped according to ploidy level (diploid, triploid, tetraploid; factor 1) or hybridity (isogenic, hybrid; factor 2)

Source	Trait	Type III Sum of Squares	df	Mean Square	F	Sig.
Ploidy	Total yield	11,600.86	2	5800.42	12.99	< 0.001
	Root yield	3418.44	2	1709.22	8.97	0.001
	Sugar content	2.00	2	1.00	3.80	0.033
	Sugar yield	93.92	2	46.96	9.98	< 0.001
	Seed viability	10,781.19	2	5390.59	104.14	< 0.001
Hybridity	Total yield	12,437.99	1	12,437.99	27.85	< 0.001
	Root yield	6293.95	1	6293.95	33.03	< 0.001
	Sugar content	7.98	1	7.98	30.27	< 0.001
	Sugar yield	207.82	1	207.82	44.16	< 0.001
	Seed viability	3980.95	1	3980.95	76.91	< 0.001

**Table 4:** Tukey HSD results displaying significant differences between ploidy levels for important agronomic traits. Both F1 2× hybrids (EA) and (EB) and F1 3× hybrids (EAA) and (EBB) and their parent lines (EE), (AA), (BB), (AAAA) and (BBBB) were analyzed according to ploidy level (diploid, triploid, tetraploid)

Trait	(I) Ploidy	(J) Ploidy	Mean Difference (I-J)	Std. Error	Sig.
Total yield	Diploid	Triploid	−16.58	8.84	0.162
		Tetraploid	84.24	8.84	< 0.001
	Triploid	Tetraploid	100.81	10.57	< 0.001
Root yield	Diploid	Triploid	−10.70	5.77	0.169
		Tetraploid	50.67	5.77	< 0.001
	Triploid	Tetraploid	61.36	6.90	< 0.001
Sugar content	Diploid	Triploid	0.04	0.22	0.986
		Tetraploid	1.14	0.22	< 0.001
	Triploid	Tetraploid	1.11	0.26	< 0.001
Sugar yield	Diploid	Triploid	−1.59	0.91	0.202
		Tetraploid	8.66	0.91	< 0.001
	Triploid	Tetraploid	10.25	1.09	< 0.001
Seed viability	Diploid	Triploid	8.56	3.01	0.021
		Tetraploid	64.31	3.01	< 0.001
	Triploid	Tetraploid	55.75	3.60	< 0.001

### Homozygous and heterozygous tetraploid male parents produce F1 triploids with different nuclear multi-locus heterozygosity levels which exhibit largely equivalent heterosis

**Fig. 4:**
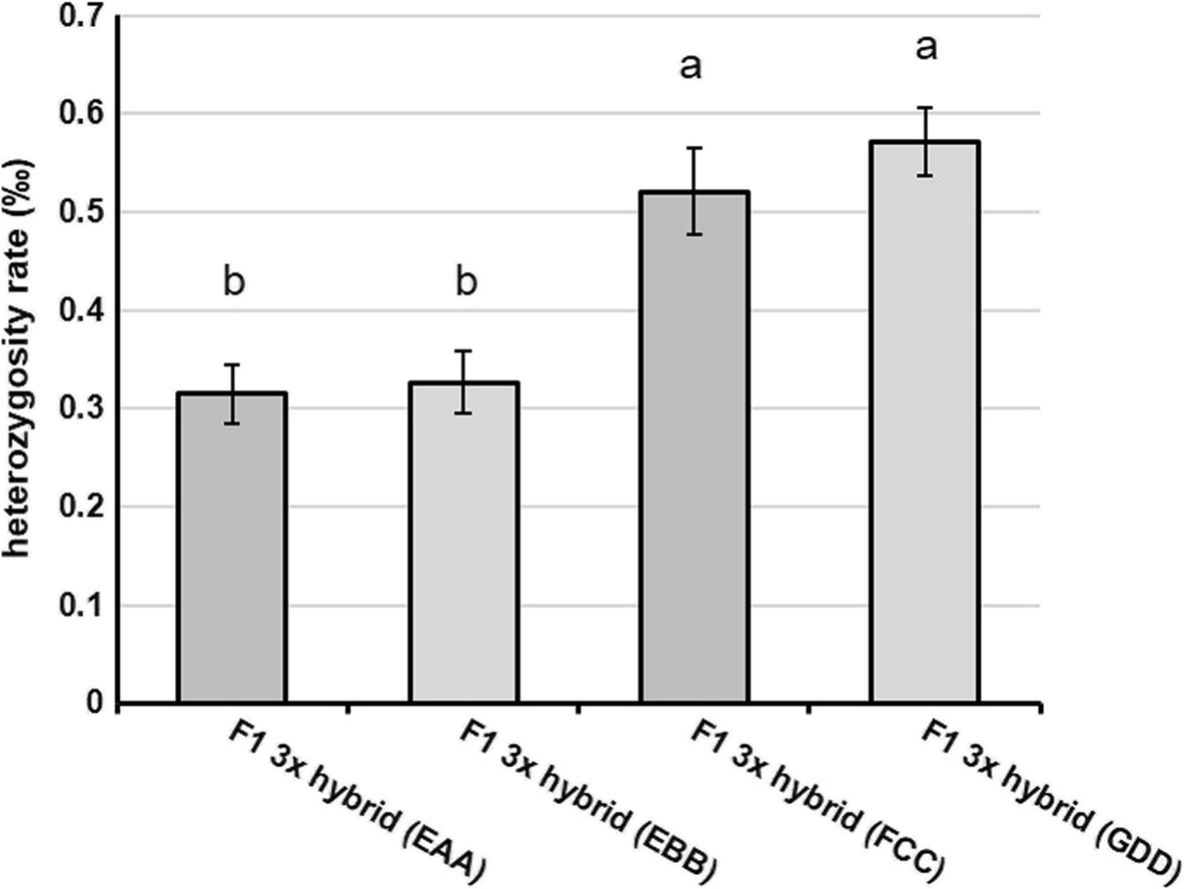
Mean genome-wide heterozygosity rate of F1 3× hybrids of sugar beet generated in this experiment. Heterozygosity rate is calculated by the ratio of heterozygous SNPs to the total number of genome bases. Data are mean of three replicates (± SD). F1 3× hybrids with heterozygous male parents, F1 3× hybrid (FCC) and (GDD), are significantly more heterozygous than F1 3× hybrids with homozygous male parents, F1 3× hybrid (EAA) and (EBB). Statistical differences were determined with a one-way ANOVA and Tukey’s HSD test. Means assigned different letters are statistically different (*P* <  0.05)

To investigate whether increased nuclear multi-locus heterozygosity in F1 hybrids in a polyploid system could affect heterosis in sugar beet, we compared the performance of F1 triploid hybrids generated from test crosses using homozygous versus highly heterozygous male parents. To determine the effect of homozygous versus heterozygous tetraploid male parent on F1 triploid heterosis, a set of F1 triploid hybrids were analysed: F1 3× hybrid (EAA) and (EBB) share the same female parent, 2× (EE), and have homozygous tetraploid male parents derived from doubled haploidy and chromosome doubling, 4× (AAAA) and (BBBB), while F1 3× hybrid (FCC) and (GDD) have different female parents, 2× (FF) and (GG), and highly heterozygous tetraploid male parents, 4× (CCCC) and (DDDD) (Table 1 and 2). The F1 3× hybrid (FCC) and (GDD) have a higher extent of multi-locus nuclear heterozygosity than the F1 3× hybrid (EAA) and (EBB), as confirmed through genotyping-by-sequencing (Fig. 4).

### F1 triploid hybrids with heterozygous tetraploid male parents exhibit both positive and negative heterosis effects on seed traits

**Fig. 5:**
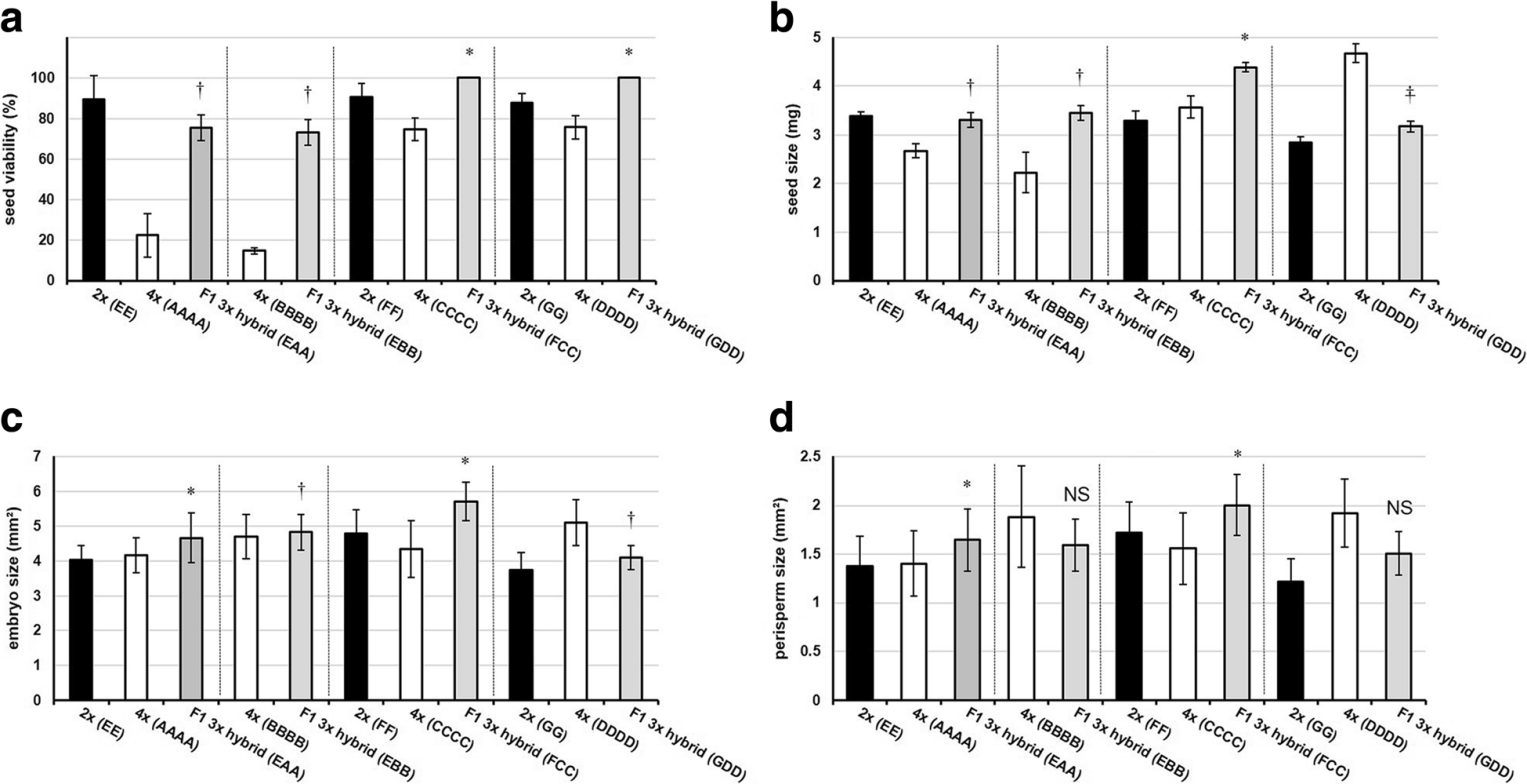
Sugar beet fruit and seed characteristics of F1 triploid hybrids and their parent lines. Data are mean of four replicates (± SD). **a** Seed viability, **b** seed size, **c** embryo size, **d** perisperm size**.** Different genotypes specified in parentheses. * Best parent heterosis (P ≤ 0.05), † Mid-parent heterosis (P ≤ 0.05), NS Not significantly different (P > 0.05), ‡ Below mid-parent value (P ≤ 0.05)

Our results indicate that 100% of the highly heterozygous F1 3× hybrid (FCC) and (GDD) fruits are monogerm and these fruits contain 100% viable seeds, indicating heterosis for seed viability (*P* ≤ 0.05) (Fig. 5a, Additional file 2: Table S3). The F1 3× hybrid (FCC) displays positive heterosis (*P* ≤ 0.05) for seed size, while the F1 3× hybrid (GDD) displays negative heterosis (*P* ≤ 0.05) for seed size (Fig. 5b). The F1 3× hybrid (FCC) displays heterosis (*P* ≤ 0.05) for embryo size. In contrast, the F1 3× hybrid (GDD) displays negative heterosis (*P* ≤ 0.05) for embryo size (Fig. 5c). Similarly, the F1 3× hybrid (FCC) displays heterosis (*P* ≤ 0.05) for perisperm size but F1 3× hybrid (GDD) has a perisperm size not significantly different (*P* = 0.10) from the mid-parent value (Fig. 5d).

### F1 triploid hybrids with heterozygous tetraploid male parents do not exhibit a uniform heterotic response in elation to root morphology, yield and sugar yield

**Fig. 6:**
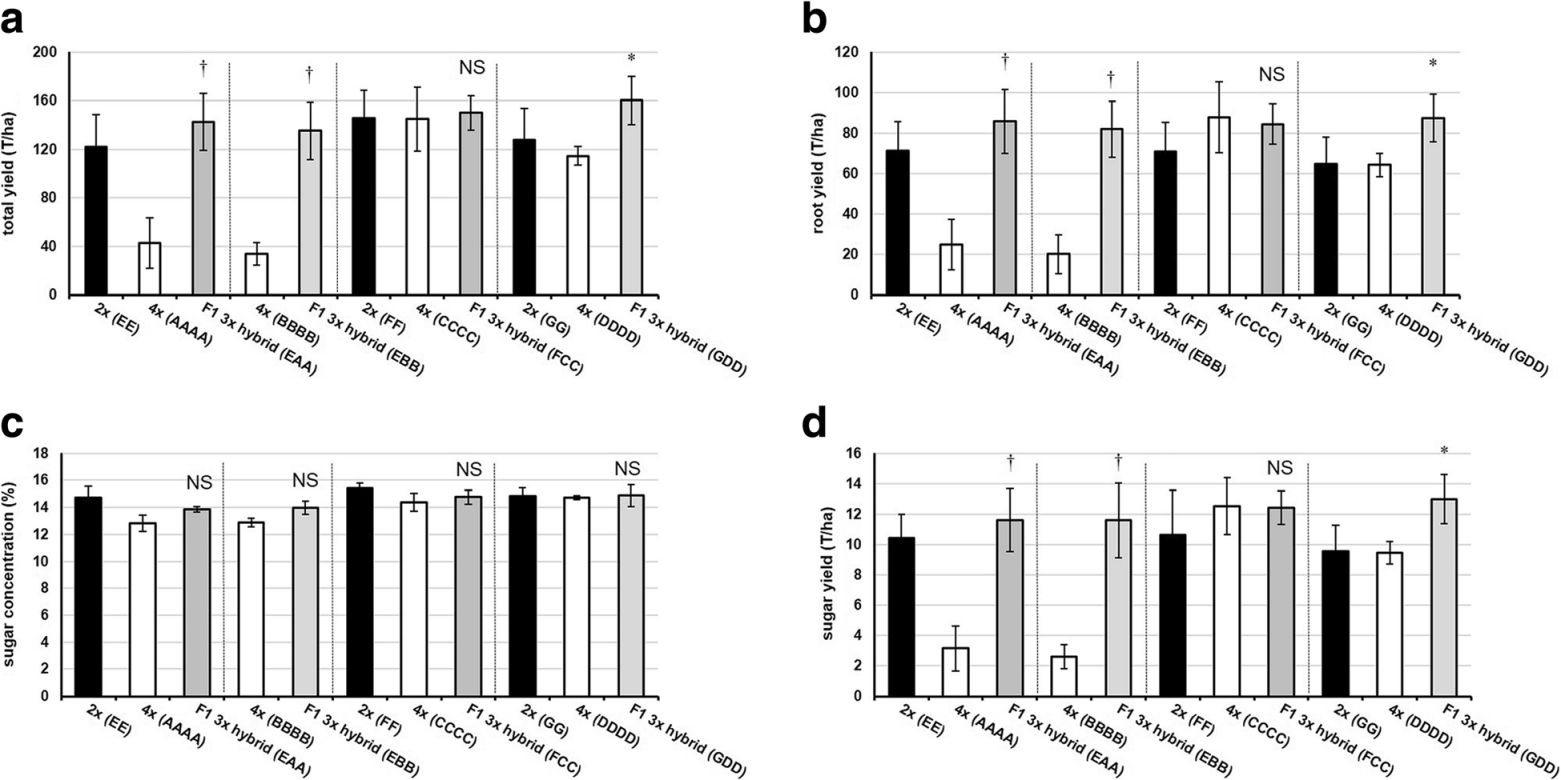
Agronomic and root quality traits of F1 triploid hybrids of sugar beet and their parental lines. Data are mean of four replicates (± SD). **a** Total yield, **b** root yield, **c** corrected sugar content, **d** corrected sugar yield. Different genotypes specified in parentheses. * Best parent heterosis (P ≤ 0.05), † Mid-parent heterosis (P ≤ 0.05), NS Not significantly different (P > 0.05)

The F1 3× hybrid (FCC) has a total yield and root yield not significantly different (*P* = 0.37, *P* = 0.31) than the mid-parent value, whereas F1 3× hybrid (GDD) displays heterosis for total yield and root yield (*P* ≤ 0.05) (Fig. 6a and b). The heterozygous male parents, 4× (CCCC) and (DDDD), both have a harvest plant density of 44.75 (Additional file 2: Table S4), the highest recorded for any male parent in this experiment. The corresponding F1 3× hybrids have a similar harvest plant density to their mid-parent values (*P* > 0.05). For above-ground biomass, both the F1 3× hybrid (FCC) and F1 3× hybrid (GDD) do not significantly differ from their mid-parent values (*P* > 0.05). Both the F1 3× hybrid (FCC) and F1 3× hybrid (GDD) show no significant difference (*P* > 0.05) in root length or root circumference from their mid-parent values (Additional file 2: Table S4). The corrected sugar content for all F1 3× hybrids does not significantly differ from their mid-parent values (*P* > 0.05) (Fig. 6c). The F1 3× hybrid (FCC) does not have a significantly different corrected sugar yield than the mid-parent value (*P* = 0.31). In contrast, the F1 3× hybrid (GDD) displays heterosis for corrected sugar yield (*P* ≤ 0.05) (Fig. 6d).

### Triploid F1 hybrids do not differ for several important agronomic traits

To determine if there is a difference between triploid F1 hybrids with homozygous and heterozygous tetraploid male parents for important agronomic traits, a one-way ANOVA was performed to compare all four F1 triploid hybrids. A post-hoc Tukey’s HSD test revealed means which are significantly different from one another. Seed viability is significantly different between groups (*P* ≤ 0.05). The F1 3× hybrid (FCC) and F1 3× hybrid (GDD) have greater seed viability than F1 3× hybrid (EAA) and F1 3× hybrid (EBB). However, total yield, root yield, corrected sugar content, and corrected sugar yield are not significantly different (*P* > 0.05) (Table 5 and Table 6).

**Table 5:** One-way ANOVA results displaying significant differences between F1 3× hybrids (EAA), (EBB), (FCC), and (GDD) for important agronomic traits

Trait	Source of variation	Sum of Squares	df	Mean Square	F	Sig.
Total yield	Between Groups	1358.47	3	452.82	1.06	0.401
	Within Groups	5111.07	12	425.92	−	
	Total	6469.54	15	−		
Root yield	Between Groups	64.29	3	21.423	0.13	0.942
	Within Groups	2019.70	12	168.31	−	
	Total	2083.99	15	−		
Sugar content	Between Groups	3.04	3	1.01	2.69	0.093
	Within Groups	4.51	12	0.38	−	
	Total	7.56	15	−		
Sugar yield	Between Groups	5.26	3	1.75	0.43	0.734
	Within Groups	48.81	12	4.07	−	
	Total	54.07	15	−		
Seed viability	Between Groups	2638.25	3	879.412	44.37	< 0.001
	Within Groups	237.83	12	19.82	−	
	Total	2876.08	15	−

**Table 6:** Tukey HSD result displaying significant differences between F1 3× hybrids (EAA), (EBB), (FCC), and (GDD) for seed viability

Trait	(I) Genotype	(J) Genotype	Mean Difference (I-J)	Std. Error	Sig.
Seed viability	F1 3× hybrid (EAA)	F1 3× hybrid (EBB)	2.28	3.15	0.886
		F1 3× hybrid (FCC)	−24.49	3.15	< 0.001
		F1 3× hybrid (GDD)	−24.49	3.15	< 0.001
	F1 3× hybrid (EBB)	F1 3× hybrid (FCC)	−26.77	3.15	< 0.001
		F1 3× hybrid (GDD)	−26.77	3.15	< 0.001
	F1 3× hybrid (FCC)	F1 3× hybrid (GDD)	0.00	3.15	1.000

Different genotypes specified in parentheses

## Discussion

### Increases in paternal genome dosage in F1 hybrids of sugar beet does not significantly enhance heterosis effects

In this study diploid and triploid F1 hybrids of sugar beet were generated which had either one paternally inherited nuclear genome set (i.e. EA, EB) or two paternally inherited nuclear genome sets (i.e. EAA, EBB) (Table 2). When the male sterile diploid line (Genotype EE) is crossed with pollen from a diploid homozygous doubled haploid (DH, Genotype AA), significant heterosis effects are seen in the diploid F1 hybrid (EA): mid-parent heterosis is regularly seen and in some instances best-parent heterosis (Fig. 2 and Fig. 3). However, when a genetically identical homozygous tetraploid pollen donor is used in the same cross (i.e. AAAA) there is no additional effect on heterosis in the triploid F1 hybrids (EAA) generated (Fig. 2, Fig. 3 and Table 3). Similarly, when a different diploid homozygous doubled haploid (DH, Genotype BB) is used, there is again no differential heterosis effect observed due to paternal genome dosage increase in the F1 hybrid triploid (EBB) relative to the F1 hybrid diploid (EB).

Our results indicate a greater impact of hybridity over paternal genome dosage on heterosis effects for important agronomic traits in sugar beet (Table 3 and Table 4). Indeed, a paternal genome dosage increase effect is not statistically significant for total yield, root yield, and sugar yield in the pooled comparison of diploid and triploid lines of genotypes (A) and (B), suggesting that the difference between them is mainly due to the effects of hybridity. The only exception is seed viability, where the effect of paternal genome dosage increase is significant between diploids and triploids (*P* ≤ 0.05) (Table 4): both F1 2× hybrids (EA) and (EB) have ~ 23% greater seed viability than their equivalent F1 3× hybrids (EAA) and (EBB), indicating a negative consequence of paternal genome dosage increase in these F1 hybrids for this trait (Fig. 2).

Investigations into the effects of genome addition in sugar beet hybrids were conducted in the early literature [44]. In such studies, eight diploid inbred lines were converted to tetraploids, and crosses were performed to generate diploid and reciprocal triploid F1 hybrids. It was concluded that diploid and triploid F1 hybrids were largely equivalent with regards to root yield and sucrose content. However, the authors indicated that their study had experimental design issues such as low hybridization success, the absence of CMS lines, and poor field germination. Using the same inbred diploid and tetraploid lines, it was later reported that the resulting diploid and reciprocal triploid hybrids were “low-producing” and “not high yielding”, i.e. there was no heterosis [45]. Another study, consisting of 120 hybrids, reported that F1 triploid hybrids averaged a 9% higher root yield than F1 diploid hybrids [46]. However, the lines used in this study were genetically diverse: the 15 male parents originated from different European countries. In such studies, it was not possible to disaggregate genome dosage effects from hybridity effects on heterosis in sugar beet. In contrast, using our crossing design it is possible to investigate paternal genome dosage versus hybridity contributions to heterosis effects, through the use of the same CMS line, equivalent homozygous double haploid (AA, BB) male parents and homozygous tetraploid (AAAA, BBBB) male parents, reliable hybridization, and field emergence of F1 hybrids and parental lines. Our results indicate that increasing paternal genome dosage does not enhance heterosis in sugar beet F1 hybrids. Indeed, we demonstrate that for some genotypes there is a negative heterosis effect on seed viability in sugar beet F1 hybrids.

Parental genome dosage effects on heterosis have previously been investigated in maize [19]. Using the maize inbred lines B73 and Mo17, diploid F1 hybrids were generated, while a trifluralin procedure was used to generate triploid F1 hybrids with two paternally derived genome sets. The diploid versus triploid F1 hybrids differed only in relation to paternal genome dosage: reciprocal F1 diploid hybrids inherited one genome from each parent, while the F1 triploid hybrids inherited either one maternal genome from B73 and two paternal genomes from Mo17, or, one maternal genome from Mo17 and two paternal genomes from B73. Analogous to our results in sugar beet, the F1 diploid and F1 triploid maize hybrids (with two paternally derived genome sets) exhibit largely equivalent heterosis over their parents, e.g. the F1 diploid and F1 triploid hybrids displayed equivalent mid-parent heterosis for plant height, leaf length and no. of tassel branches [19]. The maize F1 triploid hybrids showed a different heterotic response for a number of agronomic traits relative to their corresponding F1 diploid hybrids, e.g. F1 triploid hybrids had reduced ear length and took a longer period to reach anther emergence [19]. Unlike our study however, the authors were able to perform reciprocal crosses at the diploid level, thus allowing them to differentiate parent-of-origin effects from genome dosage effects. One of the F1 triploid hybrids exhibited higher levels of heterosis than the other F1 triploid hybrid, while the reciprocal F1 diploid hybrids displayed equal levels of heterosis [19]. The authors concluded that the difference between F1 triploid hybrids is not due to a parent-of-origin effect but rather due to genome dosage effects that can depend on the genotype in question [19].

Increasing the paternal genome dosage in *A. thaliana* has revealed a paternal genome dosage effect on heterosis for seed size and leaf area [11, 17]. Unlike maize, this is not a genotype-dependent genome dosage effect, as the paternal genome dosage effects on heterosis have been demonstrated across multiple genotypes, where F1 triploid hybrids with two paternal genomes have larger seeds and leaf area than F1 diploid hybrids [11, 17]. In our experiments with sugar beet, a genotype-dependent paternal genome dosage effect on F1 hybrids was not observed.

### Increasing heterozygosity in F1 triploid hybrids does not enhance heterosis effects

Heterozygosity is one measure of genetic variation. At a given locus in a plant genome, heterozygosity refers to the presence of different alleles for the same gene (e.g. if diploid parents are homozygous for different pairs of the same gene, say *A*_*1*_/*A*_*1*_ and *A*_*2*_/*A*_*2*_, their offspring will inherit allele *A*_*1*_ and allele *A*_*2*_ and display heterozygosity at this locus, *A*_*1*_/*A*_*2*_). Thus, the level of heterozygosity at all nuclear gene loci cumulatively determines the extent of multi-locus heterozygosity for any genotype. Genotyping-by-sequencing (GBS) analysis can be used to identify single nucleotide polymorphisms (SNPs) across the nuclear genome, and has been used to calculate rates of multi-locus heterozygosity of genotypes in cotton [47], soybean (*Glycine max*) [48], *Miscanthus sinensis* [49], maize [50], and yams (*Dioscorea*) [51].

The F1 3× hybrids generated in this study have different tetraploid male parents. The homozygous 4× (AAAA) and 4× (BBBB) genotypes have been generated by spontaneous chromosome doubling during double-haploid production and each have four identical sets of chromosomes that are homozygous. In contrast, the 4× (CCCC) and 4× (DDDD) genotypes are highly heterozygous lines (i.e. C^1^C^2^C^3^C^4^ and D^1^D^2^D^3^D^4^) used in sugar beet breeding. The resulting F1 3× hybrids can therefore paternally inherit either two homozygous genomes (to generate F1 3× hybrids (EAA) and (EBB)) or two heterozygous genomes (to generate F1 3× hybrids (FCC) and (GDD)). As a result, the heterozygosity rate (i.e. the ratio of heterozygous SNPs to the total number of genome bases) is higher for the F1 3× hybrids (FCC) and (GDD), when compared to the F1 3× hybrids (EAA) and (EBB) (Fig. 4).

The one-way ANOVA and post-hoc test results (Table 5 and Table 6) reveal that the F1 3× hybrids are similar for the majority of traits measured in the field. The only difference observed between them is for seed viability, where the fruits of F1 3× hybrid (FCC) and (GDD) show improved seed viability (Table 6). Due to the unrelated parents of the F1 3× hybrids used in this experiment it is difficult to determine whether increased nuclear heterozygosity or genotype is responsible for this effect, although it is noteworthy that the fruits of F1 3× hybrids (FCC) and (GDD) are both 100% monogerm which could be a contributing factor (Additional file 2: Table S3).

Our findings indicate that there may be no positive relationship between levels of nuclear multi-locus heterozygosity and heterosis effects for traits such as total yield and root yield in sugar beet. Some researchers have found that higher levels of multi-locus heterozygosity in oilseed rape (*Brassica napus*) leads to greater heterosis i.e. the more genetically divergent the parent lines, the greater the expression of heterosis in intraspecific crosses [26, 27]. However, in another study in oilseed rape no relationship between genetic distance and heterosis was found [28]. Likewise, there is conflicting evidence in maize, where early studies suggested heterosis increases with increasing parental genetic distance up to a certain threshold [20], but recent investigations have not found a correlation between extent of heterosis effects and parental genetic distance in maize [21–23]. A significant body of research has concluded that genetic distance does not correlate well with heterosis for intraspecific crosses, as seen in bread wheat (*Triticum aestivum)* [31], rice (*Oryza sativa*) [30], and pepper (*Capsicum annum*) [29]. Our findings in sugar beet (albeit based on a limited number of parental genotypes) suggest that there is no axiomatic relationship between parental genetic distance (and corresponding multi-locus heterozygosity levels) and heterosis effects for the agronomic traits analyzed.

### Genome dosage effects on sugar beet seed biology

The seed biology of sugar beet differs from the cereal crop maize and the model organism *A. thaliana* (Table 7). In sugar beet seed development, the maternal nucellus is not fully digested during maturation and leads to the perisperm tissue which accumulates starch reserves [52]. The perisperm starch reserve plays a nutritive role in relation to seed germination and early (approx. first 7 days) growth [53–55]. While sugar beet seeds arising from crosses between diploid parents contain triploid endosperm tissue (which is a fertilization product), the endosperm tissue is not as extensive as the diploid maternal perisperm tissue [52]. In maize and *A. thaliana* the main nutritive source for the seed embryo is the endosperm tissue, which is persistent in maize and transient in *A. thaliana* [56, 57]. Paternal genome dosage increases can only directly impact on the fertilization products (i.e. the embryo and endosperm) and not on the maternal perisperm (Table 7). If the maternal perisperm is the main nutritive tissue supporting embryo development in sugar beet this could explain why paternal genome dosage heterosis effects are not evident in sugar beet. In maize and *A. thaliana* seed, in contrast, the main nutritive tissue (the endosperm) genome dosage can be increased from 2m:1p in F1 diploids to 2m:2p in F1 triploids, with greater potential for genome dosage effects on the main nutritive tissue supporting embryo growth and development.

**Table 7:** Parental genome dosage of seed tissue in F1 diploid and F1 triploid hybrids generated in this experiment

Tissue	♀ ♂	♀ ♂
−	2× *X* 2×	2× *X* 4×
Embryo	1m:1p	1m:2p
Endosperm	2m:1p	2m:2p
Perisperm	2m:0p	2m:0p

*m* maternal genome contribution; *p* paternal genome contribution

### Hybridity can trigger increased root size without reductions in sugar concentrations in sugar beet

Heterosis breeding in sugar beet is challenged by the inverse relationship between root size and sugar content. Many studies have shown that varieties with a high root yield have low sucrose concentrations [58–60]. An ideal sugar beet variety is one that combines high root yield with high sucrose concentration (plus other characteristics such as low levels of impurities and strong abiotic and biotic stress tolerance). Our study demonstrates that there is no heterotic effect on sugar concentration in the sugar beet F1 hybrids (Fig. 3c and Fig. 6c). The moderate negative relationship (Pearson’s correlation coefficient, *r* = − 0.47) between root size and sugar concentration in a pooled analysis of all F1 hybrids suggests that root size can be increased while sugar concentration remains stable. The most extreme example of this is F1 2× hybrid (EA) which has a root yield of ~ 32 T/ha greater than that of the best parent, while the sucrose concentration has not changed (Fig. 3c). The identification of the molecular mechanisms which can allow increased root size without reductions in sugar concentrations could provide new avenues for sustainable intensification by increasing sugar yield per unit area.

## Conclusions

Our results demonstrate that increasing the paternal genome dosage in F1 triploid hybrids of sugar beet does not enhance heterosis effects beyond what can be achieved in F1 diploid hybrids. Furthermore, increasing the extent of paternally inherited nuclear multi-locus heterozygosity in F1 triploid hybrids also does not enhance the heterosis effect, suggesting there is no axiomatic relationship between heterozygosity levels and heterosis in sugar beet F1 triploids. Our results suggest that heterosis gains for important agronomic traits (e.g. root yield, sugar yield) in sugar beet can largely be achieved at the diploid breeding level.

## Additional files

Additional file 1: Detailed methods of genotyping-by-sequencing analysis. The steps taken for library preparation, high-throughput DNA sequencing, bioinformatic analysis of sequencing data, and software used are explained herein. (DOCX 370 kb).

Additional file 2: **Table S1.** Fruit and seed characteristics of F1 diploid and triploid hybrids and parent lines. Data are mean of four replicates (± SE). **Table S2.** Agronomic and root quality traits of F1 diploid and triploid hybrids and their parent lines. Data are mean of four replicates (± SE). **Table S3.** Fruit and seed characteristics of F1 triploid hybrids and parent lines. Data are mean of four replicates (± SE). **Table S4.** Agronomic and root quality traits of F1 triploid hybrids and their parent lines. Data are mean of four replicates (± SE). (DOCX 25 kb).

Additional file 3: Full data set tables. All data recorded in analysis of seed and fruit traits, seed cross-sections, and agronomic and root quality traits are displayed herein. (XLSX 94 kb).

Additional file 4: **Fig. S4.1.** Top and bottom view of representative alive and dead sugar beet seeds. **Fig. S4.2.** Flow cytometry analysis of nuclei from leaves confirms ploidy level of each line. **Fig. S4.3.** Germination test shows F1 diploid hybrids of sugar beet display heterosis effects on seed germination. Germination test shows there is a heterosis effect on germination in F1 3× hybrid (EAA) but not F1 3× hybrid (EBB). **Fig. S4.4.** F1 triploid hybrids with heterozygous tetraploid male parents display mid-parent heterosis in relation to early germination. (DOCX 7935 kb).

## Abbreviations

ANNOVAR: Annotate variation
ANOVA: Analysis of variance
BAM: Binary alignment map
BWA: Burrows-wheeler aligner
CASAVA: Consensus assessment of sequence and variation
CMS: Cytoplasmic male sterile
CSC: Corrected SC
DH: Double haploid
DNA: Deoxyribonucleic acid
F1: Filial 1
Gbit: Gigabit
gDNA: genomic DNA
InDels: Insertions or deletions in genome bases
K: Potassium
Na: Sodium
OPA: Ortho-phthaldialdehyde
PVP: Polyvinylpyrrolidone
qPCR: Quantitative real-time PCR
SC: Sugar content
SFL: Standard factory loss
SML: Standard molasses loss
SNP: Single nucleotide polymorphism
T/ha: Tons per hectare
Tukey’s HSD: Tukey’s Honest Significant Difference
α-amino N: Alpha-amino Nitrogen

## References

[1] Schnable PS, Springer NM (2013). Progress toward understanding heterosis in crop plants. Annu Rev Plant Biol.

[2] Comai L (2005). The advantages and disadvantages of being polyploid. Nat Rev Genet.

[3] Paterson AH, Wendel JF (2015). Unraveling the fabric of polyploidy. Nat Biotechnol.

[4] Roulin A, Auer PL, Libault M, Schlueter J, Farmer A, May G, Stacey G, Doerge RW, Jackson SA (2013). The fate of duplicated genes in a polyploid plant genome. Plant J:Cell Mol Bio.

[5] Tan C, Pan Q, Cui C, Xiang Y, Ge X, Li Z (2016). Genome-wide gene/genome dosage imbalance regulates gene expressions in synthetic Brassica napus and derivatives (AC, AAC, CCA, CCAA). Front Plant Sci.

[6] Washburn JD, Birchler JA (2014). Polyploids as a “model system” for the study of heterosis. Plant Reprod.

[7] Chen ZJ (2010). Molecular mechanisms of polyploidy and hybrid vigor. Trends Plant Sci.

[8] Ryder P, McKeown PC, Fort A, Spillane C. Epigenetics and heterosis in crop plants. In: Epigenetics in plants of agronomic importance: fundamentals and applications. Cham: Springer; 2014. p. 13–31.

[9] Jiang K, Liberatore KL, Park SJ, Alvarez JP, Lippman ZB (2013). Tomato yield heterosis is triggered by a dosage sensitivity of the florigen pathway that fine-tunes shoot architecture. PLoS Genet.

[10] Wang J, Tian L, Lee H-S, Wei NE, Jiang H, Watson B, Madlung A, Osborn TC, Doerge R, Comai L (2006). Genomewide nonadditive gene regulation in Arabidopsis allotetraploids. Genetics.

[11] Fort A, Ryder P, McKeown PC, Wijnen C, Aarts MG, Sulpice R, Spillane C (2016). Disaggregating polyploidy, parental genome dosage and hybridity contributions to heterosis in Arabidopsis thaliana. New Phytol.

[12] Fort A, Tuteja R, Braud M, McKeown PC, Spillane C (2017). Parental-genome dosage effects on the transcriptome of F1 hybrid triploid embryos of Arabidopsis thaliana. Plant J: Cell Mol Bio.

[13] Donoghue MT, Fort A, Clifton R, Zhang X, McKeown PC, Voigt-Zielinksi ML, Borevitz JO, Spillane C (2014). C(m)CGG methylation-independent parent-of-origin effects on genome-wide transcript levels in isogenic reciprocal F1 triploid plants. DNA Res.

[14] Auger, D.L., Gray, A.D., Ream, T.S., Kato, A., Coe, E.H. and Birchler, J.A., 2005. Nonadditive gene expression in diploid and triploid hybrids of maize. Genetics, 169(1), pp.389-397. 10.1534/genetics.104.032987 PMC1448873 15489529

[15] Donoghue MT, Fort A, Clifton R, Zhang X, McKeown PC, Voigt-Zielinksi M, Borevitz JO, Spillane C (2013). CmCGG methylation-independent parent-of-origin effects on genome-wide transcript levels in isogenic reciprocal F1 triploid plants. DNA Res.

[16] Duszynska D, McKeown PC, Juenger TE, Pietraszewska-Bogiel A, Geelen D, Spillane C (2013). Gamete fertility and ovule number variation in selfed reciprocal F1 hybrid triploid plants are heritable and display epigenetic parent-of-origin effects. New Phytol.

[17] Miller M, Zhang C, Chen ZJ (2012). Ploidy and hybridity effects on growth vigor and gene expression in Arabidopsis thaliana hybrids and their parents. G3: Genes, Genomes, Genetics.

[18] Guo M, Davis D, Birchler JA (1996). Dosage effects on gene expression in a maize ploidy series. Genetics.

[19] Yao H, Dogra Gray A, Auger DL, Birchler JA (2013). Genomic dosage effects on heterosis in triploid maize. Proc Natl Acad Sci.

[20] Moll R, Lonnquist J, Fortuno JV, Johnson E (1965). The relationship of heterosis and genetic divergence in maize. Genetics.

[21] Reif JC, Fischer S, Schrag TA, Lamkey KR, Klein D, Dhillon BS, Utz HF, Melchinger AE (2010). Broadening the genetic base of European maize heterotic pools with US Cornbelt germplasm using field and molecular marker data. Theor Appl Genet.

[22] Frisch M, Thiemann A, Fu J, Schrag TA, Scholten S, Melchinger AE (2010). Transcriptome-based distance measures for grouping of germplasm and prediction of hybrid performance in maize. Theor Appl Genet.

[23] Benchimol LL, de Souza CL, Garcia AAF, Kono PMS, Mangolin CA, Barbosa AMM, Coelho ASG, de Souza AP (2000). Genetic diversity in tropical maize inbred lines: heterotic group assignment and hybrid performance determined by RFLP markers. Plant Breed.

[24] Meyer RC, Törjék O, Becher M, Altmann T (2004). Heterosis of biomass production in Arabidopsis. Establishment during early development. Plant Physiol.

[25] Stokes D, Morgan C, O’Neill C, Bancroft I (2007). Evaluating the utility of Arabidopsis thaliana as a model for understanding heterosis in hybrid crops. Euphytica.

[26] Ali M, Copeland LO, Elias SG, Kelly JD (1995). Relationship between genetic distance and heterosis for yield and morphological traits in winter canola (*Brassica napus* L.). Theor Appl Genet.

[27] Riaz A, Li G, Quresh Z, Swati MS, Quiros CF (2001). Genetic diversity of oilseed Brassica napus inbred lines based on sequence-related amplified polymorphism and its relation to hybrid performance. Plant Breed.

[28] Diers B, McVetty P, Osborn T (1996). Relationship between heterosis and genetic distance based on restriction fragment length polymorphism markers in oilseed rape (Brassica napus L.). Crop Sci.

[29] Geleta L, Labuschagne M, Viljoen C (2004). Relationship between heterosis and genetic distance based on morphological traits and AFLP markers in pepper. Plant Breed.

[30] Zhang Q, Gao Y, Maroof MS, Yang S, Li J (1995). Molecular divergence and hybrid performance in rice. Mol Breed.

[31] Martin J, Talbert L, Lanning S, Blake N (1995). Hybrid performance in wheat as related to parental diversity. Crop Sci.

[32] Draycott AP. Sugar beet. New York: Wiley; 2008.

[33] Peto F, Boyes J (1940). Comparison of diploid and triploid sugar beets. Can J Res.

[34] Savitsky V. Monogerm sugar beets in the United States. Proc Amer Soc Sugar Beet Tech. 1950;6:156–9.

[35] Biancardi E, McGrath JM, Panella LW, Lewellen RT, Stevanato P. Sugar beet. In: Root and tuber crops: Springer; New York, NY. 2010. p. 173–219.

[36] Hedtke SM, Hillis DM (2011). The potential role of Androgenesis in cytoplasmic–nuclear phylogenetic discordance. Syst Biol.

[37] Van Dyke F. Conservation biology: foundations, concepts, Applications: Springer Science & Business Media; 2008.

[38] Li H, Durbin R (2009). Fast and accurate short read alignment with burrows–wheeler transform. Bioinformatics.

[39] Li H, Handsaker B, Wysoker A, Fennell T, Ruan J, Homer N, Marth G, Abecasis G, Durbin R (2009). The sequence alignment/map format and SAMtools. Bioinformatics.

[40] Wang K, Li M, Hakonarson H (2010). ANNOVAR: functional annotation of genetic variants from high-throughput sequencing data. Nucleic Acids Res.

[41] Kenter C, Hoffmann C, Märländer B (2006). Sugarbeet as raw material–advanced storage management to gain good processing quality/Optimierung der Rohstoffqualität von Zuckerrüben durch verbessertes Lagerungsmanagement. Zuckerindustrie.

[42] De Whalley HCS. ICUMSA methods of sugar analysis: official and tentative methods recommended by the International Commission for Uniform Methods of sugar analysis (ICUMSA): Elsevier; Amsterdam 2013.

[43] Buchholz K, Märländer B, Puke H, Glattkowski H, Thielecke K. Neubewertung des technischen Wertes von Zuckerrueben. Zuckerindustrie. 1995;120(2), pp.113–121.

[44] Hecker R, Stafford R, Helmerick R, Maag G (1970). Comparison of the same sugarbeet F1 hybrids as diploids, triploids and tetraploids. J Am Soc Sugar Beet Technol.

[45] Smith G, Hecker R, Martin S (1979). Effects of ploidy level on the components of sucrose yield and quality in sugarbeet. Crop Sci.

[46] Lasa J, Romagosa I, Hecker R, Sanz J (1989). Combining ability in diploid and triploid sugarbeet hybrids from diverse parents. J Sugar Beet Res.

[47] Islam MS, Thyssen GN, Jenkins JN, Fang DD. Detection, validation, and application of genotyping-by-sequencing based single nucleotide polymorphisms in upland cotton. Plant Gen. 2015;8(1):1–10 10.3835/plantgenome2014.07.0034 33228292

[48] Sonah H, Bastien M, Iquira E, Tardivel A, Légaré G, Boyle B, Normandeau É, Laroche J, Larose S, Jean M (2013). An improved genotyping by sequencing (GBS) approach offering increased versatility and efficiency of SNP discovery and genotyping. PLoS One.

[49] Ma X-F, Jensen E, Alexandrov N, Troukhan M, Zhang L, Thomas-Jones S, Farrar K, Clifton-Brown J, Donnison I, Swaller T (2012). High resolution genetic mapping by genome sequencing reveals genome duplication and tetraploid genetic structure of the diploid Miscanthus sinensis. PLoS One.

[50] Azmach G, Gedil M, Menkir A, Spillane C (2013). Marker-trait association analysis of functional gene markers for provitamin a levels across diverse tropical yellow maize inbred lines. BMC Plant Biol.

[51] Girma G, Hyma KE, Asiedu R, Mitchell SE, Gedil M, Spillane C (2014). Next-generation sequencing based genotyping, cytometry and phenotyping for understanding diversity and evolution of Guinea yams. Theor Appl Genet.

[52] Hermann K, Meinhard J, Dobrev P, Linkies A, Pesek B, Heß B, Macháčková I, Fischer U, Leubner-Metzger G (2007). 1-Aminocyclopropane-1-carboxylic acid and abscisic acid during the germination of sugar beet (Beta vulgaris L.): a comparative study of fruits and seeds. J Exp Bot.

[53] Lawrence DM, Halmer P, Bowles DJ (1990). Mobilisation of storage reserves during germination and early seedling growth of sugar beet. Physiol Plant.

[54] Elamrani A, Raymond P, Saglio P (1992). Nature and utilization of seed reserves during germination and heterotrophic growth of young sugar beet seedlings. Seed Sci Res.

[55] Catusse J, Strub JM, Job C, Van Dorsselaer A, Job D (2008). Proteome-wide characterization of sugarbeet seed vigor and its tissue specific expression. Proc Natl Acad Sci U S A.

[56] Costa LM, Gutièrrez-Marcos JF, Dickinson HG (2004). More than a yolk: the short life and complex times of the plant endosperm. Trends Plant Sci.

[57] Baroux C, Spillane C, Grossniklaus U (2002). Evolutionary origins of the endosperm in flowering plants. Genome Biol.

[58] Bergen P (1967). Seasonal patterns of sucrose accumulation and weight increase in sugar beets. J Am Soc Sugar Beet Technol.

[59] Oldemeyer R (1975). Introgressive hybridization as a breeding method in Beta vulgaris. J Am Soc Sugar Beet Technol.

[60] Carter J (1987). Sucrose production as affected by root yield and sucrose concentration of sugarbeets. J Am Soc Sugar Beet Technol.

