# Supplementary material for: Hybridity has a greater effect than paternal genome dosage on heterosis in sugar beet (Beta vulgaris)
Source: BMC Plant Biol. 2018 Jun 15;18:120. doi: 10.1186/s12870-018-1338-x (PMC6003118; doi:10.1186/s12870-018-1338-x)
Supplement: Supplementary file 1 — Detailed methods of genotyping-by-sequencing analysis. The steps taken for library preparation, high-throughput DNA sequencing, bioinformatic analysis of sequencing data, and software used are explained herein. (DOCX 370 kb). [file 12870_2018_1338_MOESM1_ESM.docx]

## Library Preparation and Sequencing

Throughout the whole process of sequencing from the DNA sample to the final data, each step, including sample test, library preparation, and sequencing procedures, influences the quality of data production, while the data quality further impacts on the analysis results directly. To guarantee the accuracy and reliability of the sequencing data, we utilized stringent quality control (QC) procedures. The workflow was as follows: 1. DNA quantification and qualification, 2. Library construction, 3. Library quality control, High-throughput DNA sequencing, 5. Quality control (QC).

### 1. DNA quantification and qualification

The three major QC methods for DNA sample qualification were as follows: 1. Agarose gel electrophoresis analysis for DNA purity and integrity, 2. NanoDrop^®^ 2000 spectrophotometer measurement for DNA purity by assessing the OD_260_/OD_280_ ratio, 3. Qubit^®^ 2.0 fluorometer quantitation for accurate measurement of DNA concentration. Sample DNA, with OD_260_/OD_280_ ratio of 1.8 to 2.0 and total amount of more than 1.5 µg, was used for library construction.

### 2. Library Construction

Circa 0.3~0.6μg of gDNA was digested with restriction enzymes (xxx, xxx), and the resulting digested fragments were ligated to two barcoded adapters, the universal adapter (5’ AATGATACGGCGACCACCGAGATCTACACTCTTTCCCTACACGACGCTCTTCCGATCT 3’) and indexed adapter (5’ GATCGGAAGAGCACACGTCTGAACTCCAGTCAC‐NNNNNN‐ATCTCGTATGCCGTCTTCTGCTTG 3’) with compatible sticky end corresponding to the restriction digestion enzyme. Following several rounds PCR amplification, all the samples were pooled and size-selected for the required fragments to complete the library construction. This is because the ligation of adaptors at both ends of the DNA fragment confers different sequences at the 5’ and 3’ ends of each strand in the genomic fragment and occasionally one or both adaptors will not ligate. The ligation reaction products are purified and size‐selected by agarose gel electrophoresis. Size‐selected DNA is PCR amplified to enrich for fragments that have adapters on both ends. The experimental procedures are as follows: 1. *In/Ex silico* digestion evaluation: The genome assembly was subjected to *in silico* digestion analysis to aid the optimization of enzyme sets and fragment size, ensure even genome coverage, and identification of repeated regions. The combination of experimental digestion assay with the computational approach ensures high reliable and reproducible data production. 2. Restriction enzyme digestion: 0.3~0.6 μg genomic DNA was digested completely with the optimized restriction enzyme set, in order to obtain a suitable marker density. 3. Ligating P5 and P7 adapter: each end of digested fragment was respectively ligated with P5 and P7 barcoded-adapter (with complementarily sticky ends to the digested DNA). 4. PCR enrichment and fragment selection: tags containing both P5 and P7 adapters were amplified through PCR. After DNA fragments of different samples were pooled, the desired fragments of DNA were recovered from gel electrophoresis. 5. High-throughput DNA sequencing: After cluster preparation, high-throughput DNA sequencing was performed on Illumina HiSeq platform

The experimental procedures of DNA library preparation are shown in Figure 1.


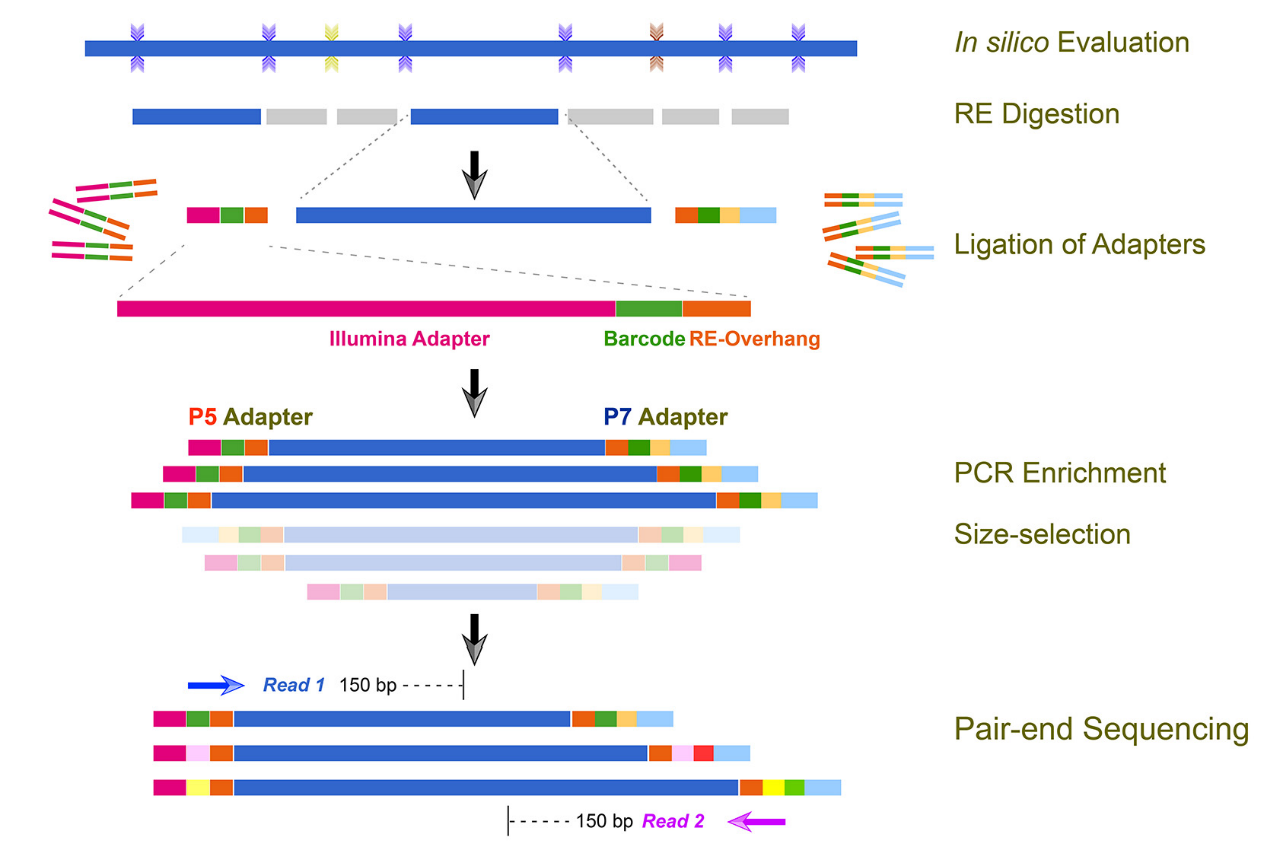


Figure 1. Experimental procedures of library preparation.

### 3. Library Quality Control

To check the prepared DNA libraries, Qubit^®^ 2.0 fluorometer was used to determine the concentration of the library. After dilution to 1 ng/ul, the Agilent^®^ 2100 bioanalyzer was used to assess the insert size. Finally, quantitative real-time PCR (qPCR) was performed to detect the effective concentration of each library. If the library with appropriate insert size has an effective concentration of more than 2 nM, the constructed libraries are of sufficient quality and ready for Illumina^®^ high-throughput sequencing.

### 4. High-throughput DNA Sequencing

The DNA libraries were pooled according to their effective concentration as well as the expected data production. Pair-end sequencing was performed on Illumina^®^HiSeq platform, with the read length of 144 bp at each end.

## Bioinformatics Analysis

The bioinformatic analysis procedures are as follows: 1. Creation of FASTQ files 2. Quality control of raw sequencing data for clean data filtration, 3. Mapping clean reads to reference genome, 4. SNP and InDel detection and annotation according to the reference genome mapping results.

### 1. Creation of FASTQ files

The original data acquired by Illumina^®^HiSeq platform in image files are firstly converted into sequence data by base calling with the CASAVA software (version 1.8). The sequences and corresponding sequencing quality information are stored in a FASTQ file.

Every read in FASTQ format is stored in four lines as follows:

@K00124:82:H2MH5BBXX:1:1101:31389:1158 2:N:0:0
TAGCCACATAGAAACCAACAGCCATATAACTGGTAGCTTTAAGCGGCTCACCTTTAGCATCAACAGGCCACAACCAACCAGAACGTGAAAAAGCGTCCTGCGTGTAGCGAACTGCGATGGGCATACAGATCGGAAGAGCGTCGTGTAGGG
+
AAFFFKKKKKKKKFKKKFFKKAAFKKKKKFKKKKFKKA,FKKKKKKKKKAKKFKKKKKKKAKKKKKKFFKKKKF<FFKKKKKKKKKKKKKFKKFKKF7FFFFFKFKKKFKKKKKKKKF<FFKKKKFKKKKKFKFKFKKFK<<F,A7,AFK

Line 1 begins with an '@' character and is followed by Illumina sequence identifiers, and an optional description (such as a FASTA title line). Line 2 is the sequence of a sequencing read. Line 3 begins with a '+' character and is optionally followed by Illumina sequence identifier and description. Line 4 encodes the quality values for the sequence in Line 2, and must contain the same number of characters as the bases in the sequence. The per base sequencing quality score could be calculated by the ASCII value of each character in Line 4 minus a constant 33.

| EAS139 | Unique instrument name |
| --- | --- |
| 136 | Run ID |
| FC706VJ | Flowcell ID |
| 2 | Flowcell lane |
| 2104 | Tile number within the flowcell lane |
| 15343 | 'x'-coordinate of the cluster within the tile |
| 197393 | 'y'-coordinate of the cluster within the tile |
| 1 | Member of a pair, 1 or 2 (paired-end or mate-pair reads only) |
| Y | Y if the read fails filter (read is bad), N otherwise |
| 18 | 0 when none of the control bits are on, otherwise it is an even number |
| ATCACG | Index sequence |

The raw sequence FASTQ files are available on the NCBI Sequence Read Archive, data set SRP125454.

### 2. Quality Control of Sequencing Data

2.1 Sequencing Quality Distribution

If the sequencing error rate is represented by e, and Illumina HiSeq^TM^ 2500 sequencing quality by Q_phred_, the quality score of a base (Phred score) is calculated by the following equation: Q_phred_ = -10log_10_(e). The correspondence relationship between Illunima sequencing quality and Phred score in base calling by CASAVA is listed as follows:

| **Phred score** | **Error Rate** | **Correct Rate** | **Q-score** |
| --- | --- | --- | --- |
| 10 | 1/10 | 90% | Q10 |
| 20 | 1/100 | 99% | Q20 |
| 30 | 1/1000 | 99.9% | Q30 |
| 40 | 1/10000 | 99.99% | Q40 |

For next-generation sequencing (NGS), the sequencing platform, chemical reactants, and sample quality can influence sequencing quality and base error rate. Sequencing quality distribution is examined over the full length of all sequences to detect any sites (base positions) with an unusually low sequencing quality and where incorrect bases may be incorporated at abnormally high levels. For detailed sequencing quality distribution, please refer to Additional File 3.

2.2 Distribution of Sequencing Errors

Sequencing error rate is related to the base quality of the obtained sequence. The sequencing platform, chemical reactants, and sample quality can all influence sequencing error rate and herein the base quality. For NGS with sequencing-by-synthesis strategy, sequencing error rate distribution shows two common features: 1. Error rate increases with extending of the sequencing reads due to the consumption of chemical reagents, damage of the DNA template by laser irradiation, and possible accumulation of errors during the sequencing cycles. All the Illumina high-throughput sequencing platforms have this feature. 2. The sequencing error rate is higher for the first several bases than at other positions, which is likely the result of reading errors during the first few cycles after calibration of the optical instruments.

Sequencing error rate distribution is examined over the full length of all sequences, to detect any sites (base positions) with an unusually high error rate, where incorrect bases may be incorporated at abnormally high levels. For detailed sequencing error distribution, please refer to Additional File 4.

2.3 Sequencing Data Filtration

Raw data obtained from sequencing contains adapter contamination and low-quality reads. These sequencing artefacts may increase the complexity of downstream analyses, and therefore, we utilize quality control steps to remove them. Consequently, all the downstream analyses are based on the clean reads. The quality control steps are as follows: 1. Discard the paired reads when either read contains adapter contamination. 2. Discard the paired reads when uncertain nucleotides (N) constitute more than 10 percent of either read. 3. Discard the paired reads when low quality nucleotides (base quality less than 5, Q ≤ 5) constitute more than 50 percent of either read. For detailed sequencing read classification, please refer to Additional File 5.

2.4 Statistics of Sequencing Data

Statistics of sequencing data are listed in Additional File 6*.*

2.5 Sequencing Evaluation Summary

In total 29.068Gbit raw data were sequenced from this run, with 29.065Gbit clean data generated after filtering low-quality data. The raw data production for each sample ranged from 519.515 M to 784.908 M, indicating a sufficient amount of data production. As the Q20 and Q30 reached 94.02% and 85.94%, respectively, the sequencing quality meets the proper analysis requirements. The GC content of 35.92% to 38.22% is also in the normal distribution range, fulfilling the quality standard.

In conclusion, the library construction and sequencing procedures are successful and highly reliable.

### 3. Mapping Statistics

The sequencing data was aligned with the reference sequence through BWA (Li and Durbin 2009) software (parameters: mem -t 4 -k 32 -M), and the mapping rate and coverage was counted according to the alignment results (see Table 4.3). The BAM files were handled by SAMtools (Li et al. 2009).

3.1　Statistics of Reference Genome

Reference genome is available at: <http://bvseq.molgen.mpg.de/Genome/Download/RefBeet-1.2/RefBeet-1.2.fna.gz>

Statistics of the reference genome are listed in _Additional File 7_.

3.2 Mapping Statistics with Reference Genome and Tag Summary

The mapping rates of samples reflect the similarity between each sample and the reference genome. The depth and coverage are indicators of the evenness and homology with the reference genome. With GBS, tag-related statistics are also calculated.

Statistics of mapping rate, depth and coverage, as well as tag-related statistics are listed in Additional File 8.

3.3 Mapping Summary

For the current 566,571,340 bp reference genome, the mapping rate of each sample ranges from 97.59% to 98.31%. The average depth on the reference genome (without Ns) is in 5.19X to 9.7X range, while the more than 1X coverage exceeds 12.17%. This result is in the qualified normal range and may serve in the subsequent variation detection and related analyses.

3.4 Enzymatic Digestion Summary

Among pairs of clean reads, those containing the exact conserved sequence of the first restriction enzyme at the beginning ends of both Read1 and Read2 are considered as successfully enzyme-catched reads, while those containing no recognition sequence of both the primary and additional restriction enzyme(s) are considered as completely cut reads. In this experiment, the ratio of enzyme-catched reads is among 97.2% to 99.3%, while the enzyme-digestion ratio ranges from 80.6% to 93.7%. All enzymatic digestion statistics are listed in Additional File 9.

### 4. SNP Detection & Annotation

Single nucleotide polymorphism (SNP) refers to a variation in a single nucleotide which may occur at some specific position in the genome, including transition and transversion of a single nucleotide. We detected the individual SNP variations using SAMtools (Li et al. 2009) with the following parameter: 'mpileup -m 2 -F 0.002 -d 1000'.

To reduce the error rate in SNP detection, we filtered the results with the criterion as follows: 1. The number of support reads for each SNP should be more than 4. 2. The mapping quality (MQ) of each SNP should be higher than 20.

4.1 Statistics of SNP Detection & Annotation

ANNOVAR (Wang et al. 2010) is a widely used software in variation annotation with multiple capabilities, including gene-based annotation, region-based annotation, filter-based annotation as well as other functionalities. We used ANNOVAR to perform annotation of detected SNPs. The UCSC known genes (https://genome.ucsc.edu/) were used for gene and region annotations.

Statistics of SNP detection and annotation are listed in Additional File 11.

4.2 SNP Quality Distribution

To assess the credibility of detected SNPs, we checked the distribution of support reads number, SNP quality, as well as the distance between adjacent SNPs.

4.3 SNP Mutation Frequency

Taking the T:A>C:G mutations as an example, this category includes mutations from T to C and A to G. When T>C mutation appears on either of the double-strand, the A>G mutation will be found in the same position of the other chain. Therefore, the T>C and A>G mutations are classified into one category. Accordingly, the whole-genome SNP mutations could be classified into six categories. The frequency of each type is shown in Figure 2.

[
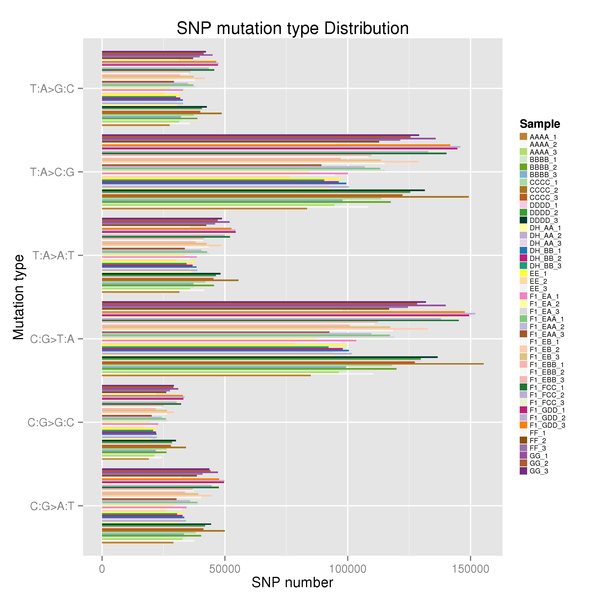
](file:///C:\Users\Brendan\GBS%20downloaded%20results\results\reports\src\images\SNP_frequency.png)

Figure 2. Frequency of SNP mutations.

The x-axis represents the number of the SNPs, and y-axis indicates the mutation types.

## References

Li, H., and R. Durbin, 2009 Fast and accurate short read alignment with Burrows–Wheeler transform. *Bioinformatics* 25 (14):1754-1760.

Li, H., B. Handsaker, A. Wysoker, T. Fennell, J. Ruan *et al.*, 2009 The sequence alignment/map format and SAMtools. *Bioinformatics* 25 (16):2078-2079.

Wang, K., M. Li, and H. Hakonarson, 2010 ANNOVAR: functional annotation of genetic variants from high-throughput sequencing data. *Nucleic acids research* 38 (16):e164-e164.

## Appendix

### List of Software

| **Analysis** | **Software** | **Usage** | **Version** |
| --- | --- | --- | --- |
| Mapping | BWA | Mapping clean reads to the reference genome and generation of bam result files. | 0.7.8-r455 |
|  | SAMtools | Sorting the bam files. | 0.1.19-44428cd |
| SNP/InDel Detection | SAMtools | Detection and filtration of SNPs and InDels. | 0.1.19-44428cd |
| Variation Annotation | ANNOVAR | Annotation of the detected variations. | 2013Aug23 |
