# Supplementary material for: Hybridity has a greater effect than paternal genome dosage on heterosis in sugar beet (Beta vulgaris)
Source: BMC Plant Biol. 2018 Jun 15;18:120. doi: 10.1186/s12870-018-1338-x (PMC6003118; doi:10.1186/s12870-018-1338-x)
Supplement: Supplementary file 2 — Table S1. Fruit and seed characteristics of F1 diploid and triploid hybrids and parent lines. Data are mean of four replicates (± SE). Table S2. Agronomic and root quality traits of F1 diploid and triploid hybrids and their parent lines. Data are mean of four replicates (± SE). Table S3. Fruit and seed characteristics of F1 triploid hybrids and parent lines. Data are mean of four replicates (± SE). Table S4. Agronomic and root quality traits of F1 triploid hybrids and their parent lines. Data are mean of four replicates (± SE). (DOCX 25 kb). [file 12870_2018_1338_MOESM2_ESM.docx]

# Supplementary Data

### Table S1. Fruit and seed characteristics of F1 diploid and triploid hybrids and parent lines. Data are mean of four replicates (± SE).

|  | ♀ | ♂ | F1  2x hybrid (EA) | ♂ | F1  3x hybrid (EAA) | ♂ | F1  2x hybrid (EB) | ♂ | F1  3x hybrid (EBB) |
| --- | --- | --- | --- | --- | --- | --- | --- | --- | --- |
|  |  |  |  |  |  |  |  |  |  |
| Trait | 2x  (EE) | 2x DH (AA) |  | 4x (AAAA) |  | 2x DH (BB) |  | 4x (BBBB) |  |
| Monogermity (%) | 89.00  (± 3.00) | 2.00  (± <0.01) | 99.04  (± 0.96) | 7.92  (± 5.44) | 50.89  (± 26.06) | 0.00  (± 0.00) | 99.00  (± 1.00) | 0.00  (± 0.00) | 77.00  (± 21.69) |
| Fruit weight (mg) | 7.45  (± 0.23) | 13.12  (± 0.59) | 7.16  (± 0.27) | 22.14  (± 0.90) | 8.50  (± 0.29) | 9.17  (± 0.35) | 6.59  (± 0.24) | 12.87  (± 0.58) | 9.96  (± 0.32) |
| Seed viability (%) | 89.26  (± 5.91) | 60.56  (± 1.10) | 98.08  (± 0.96) † | 22.49  (± 5.56) | 75.51  (± 4.44) † | 70.73  (± 2.45) | 96.00  (± 1.92) † | 14.75  (± 1.97) | 73.20  (± 3.78) † |
| Seed weight (mg) | 3.39  (± 0.08) | 2.45  (± 0.05) | 3.40  (± 0.22) † | 2.68  (± 0.14) | 3.31  (±0.16) † | 2.63  (± 0.09) | 3.15  (± 0.012) † | 2.23  (± 0.42) | 3.45  (± 0.16) † |
| Seed:Fruit weight ratio | 0.47  (± 0.02) | 0.23  (± 0.02) | 0.50  (± 0.04) * | 0.09  (± 0.01) | 0.39  (± 0.10) ^NS^ | 0.33  (± 0.02) | 0.49  (± 0.05) † | 0.18  (± 0.04) | 0.36  (± 0.02) ~~†~~ |
| Embryo size (mm^2^) | 4.04  (± 0.73) | 3.86  (± 0.94) | 4.67  (± 1.15) * | 4.17  (± 0.91) | 4.67  (± 1.31) * | 4.10  (± 1.06) | 4.42  (± 1.13) * | 4.71  (± 1.15) | 4.83  (0.95) † |
| Perisperm size (mm^2^) | 1.38  (± 0.57) | 1.20  (± 0.41) | 1.72  (± 0.53) * | 1.40  (± 0.61) | 1.64  (± 0.58) * | 1.47  (± 0.48) | 1.48  (± 0.49) ^NS^ | 1.88  (± 0.95) | 1.59  (± 0.49) ^NS^ |

DH = double haploid. Different genotypes specified in parentheses. * Best parent heterosis (*P* ≤ 0.05), † Mid-parent heterosis (*P* ≤ 0.05), ^NS^ Not significantly different (*P >* 0.05), ~~†~~ Below mid-parent value (*P* ≤ 0.05)

### Table S2. Agronomic and root quality traits of F1 diploid and triploid hybrids and their parent lines. Data are mean of four replicates (± SE).

|  | ♀ | ♂ | F1  2x hybrid (EA) | ♂ | | F1  3x hybrid (EAA) | ♂ | | F1  2x hybrid (EB) | ♂ | | F1  3x hybrid (EBB) |
| --- | --- | --- | --- | --- | --- | --- | --- | --- | --- | --- | --- | --- |
|  |  |  |  |  |  |  |  |  |  |  |  |  |
| Agronomic Traits | 2x  (EE) | 2x DH (AA) |  | 4x (AAAA) | |  | 2x DH (BB) | |  | 4x (BBBB) | |  |
| Harvest plant density ^1^ | 46.5  (± 1.71) | 39.75  (± 2.78) | 46.5  (± 2.56) ^NS^ | 19.5  (± 3.20) | | 43.75  (± 1.09) † | 43.75  (± 1.12) | | 46.5  (± 1.50) ^NS^ | 18.5  (± 4.72) | | 44.5  (± 2.06) † |
| Total yield (T/ha) | 121.79  (± 13.46) | 91.96  (± 8.68) | 166.64  (± 5.42) * | 42.84  (± 10.39) | | 142.68  (± 11.77) † | 98.75  (± 17.86) | | 140.16  (± 7.70) † | 33.68  (± 4.74) | | 135.47  (± 11.83) † |
| Above-ground biomass (g) ^2^ | 515.2  (± 63.79) | 576.04  (± 34.20) | 718.71  (± 62.34) * | 463.63  (± 58.42) | | 684.6  (± 7.79) * | 464.08  (± 58.15) | | 575.48  (± 47.20) ^NS^ | 352.13  (± 28.90) | | 608.8  (± 67.97) † |
| Root length (cm) | 17.25  (± 0.58) | 18.23  (± 1.06) | 18.73  (± 0.31) ^NS^ | 15.85  (± 0.95) | | 18.88  (± 0.60) * | 15.74  (± 0.41) | | 18.80  (± 0.80) † | 14.73  (± 0.42) | | 17.91  (± 0.61) † |
| Root circumference (cm) | 32.04  (± 1.36) | 31.42  (± 0.78) | 37.54  (± 0.65) * | 29.98  (± 1.48) | | 37.32  (± 0.65) * | 32.31  (± 1.97) | | 36.72  (± 0.73) * | 29.52  (± 1.75) | | 35.73  (± 1.24) * |
| Root yield (T/ha) | 71.44  (± 7.13) | 51.60  (± 5.84) | 103.06  (± 5.84) * | 24.87  (± 6.19) | | 85.81  (± 7.92) † | 56.06  (± 10.92) | | 88.63  (± 4.79) * | 20.24  (± 3.26) | | 82.03  (± 6.90) † |
| Root Quality Traits |  | | | |  | | |  | | |  | |
| Corrected sugar content (%) | 14.74  (± 0.40) | 13.23  (± 0.27) | 14.13  (± 0.08) ^NS^ | 12.84  (± 0.30) | | 13.87  (± 0.09) ^NS^ | 13.16  (± 0.14) | | 14.68  (± 0.10) ^NS^ | 12.87  (± 0.16) | | 13.98  (± 0.24) ^NS^ |
| Standard molasses loss (%) | 1.85  (± 0.07) | 1.99  (± 0.07) | 1.80  (± 0.03) ~~*~~ | 2.19  (± 0.07) | | 1.86  (± 0.02) ^†^ | 1.85  (± 0.07) | | 1.71  (± 0.03) ^NS^ | 1.93  (± 0.10) | | 1.81  (± 0.06) ^NS^ |
| Corrected sugar yield (T/ha) | 10.44  (± 0.78) | 6.86  (± 0.91) | 14.57  (± 0.88) * | 3.14  (± 0.74) | | 11.89  (± 1.04) † | 7.34  (± 1.36) | | 13.00  (± 0.65) * | 2.59  (± 0.40) | | 11.60  (± 1.23) † |

DH = double haploid. Different genotypes specified in parentheses. * Best parent heterosis (*P* ≤ 0.05), † Mid-parent heterosis (*P* ≤ 0.05), ^NS^ Not significantly different (*P >* 0.05), ~~*~~ Below best parent value (*P* ≤ 0.05), ~~†~~ Below mid-parent value (*P* ≤ 0.05)

### Table S3. Fruit and seed characteristics of FI triploid hybrids and parent lines. Data are mean of four replicates (± SE).

|  | ♀ | ♂ | F1  3x hybrid (EAA) | ♂ | F1  3x hybrid (EBB) | ♀ | ♂ | F1  3x hybrid (FCC) | ♀ | ♂ | F1  3x hybrid (GDD) |
| --- | --- | --- | --- | --- | --- | --- | --- | --- | --- | --- | --- |
|  |  |  |  |  |  |  |  |  |  |  |  |
| Trait | 2x  (EE) | 4x (AAAA) |  | 4x (BBBB) |  | 2x  (FF) | 4x (CCCC) |  | 2x  (GG) | 4x (DDDD) |  |
| Monogermity (%) | 89.00  (± 3.00) | 7.92  (± 5.44) | 50.89  (± 26.06) | 0.00  (± 0.00) | 77.00  (± 21.69) | 95.08  (± 0.92) | 0.00  (± 0.00) | 100  (± 0.00) | 71.00  (± 11.71) | 3.92  (± 1.57) | 100  (± 0.00) |
| Fruit weight (mg) | 7.45  (± 0.23) | 22.14  (± 0.90) | 8.50  (± 0.29) | 12.87  (± 0.58) | 9.96  (± 0.32) | 17.90  (± 0.70) | 21.14  (± 0.66) | 10.08  (± 0.21) | 9.78  (± 0.47) | 22.45  (± 0.85) | 7.81  (± 0.11) |
| Seed viability (%) | 89.26  (± 5.91) | 22.49  (± 5.56) | 75.51  (± 4.44) † | 14.75  (± 1.97) | 73.20  (± 3.78) † | 90.46  (± 2.41) | 74.84  (± 2.91) | 100  (± 0.00) * | 87.98  (± 1.49) | 75.75  (± 5.64) | 100  (± 0.00) * |
| Seed weight (mg) | 3.39  (± 0.08) | 2.68  (± 0.14) | 3.31  (± 0.16) † | 2.23  (± 0.42) | 3.45  (± 0.16) † | 3.29  (± 0.20) | 3.57  (± 0.23) | 4.39  (± 0.09) * | 2.86  (± 0.12) | 4.67  (± 0.20) | 3.17  (± 0.11) ~~†~~ |
| Seed:Fruit weight ratio | 0.47  (± 0.02) | 0.09  (± 0.01) | 0.39  (± 0.10) ^NS^ | 0.18  (± 0.04) | 0.36  (± 0.02) ~~†~~ | 0.15  (± 0.01) | 0.22  (± 0.01) | 0.44  (± <0.01) * | 0.35  (± 0.01) | 0.23  (± 0.04) | 0.52  (± <0.01) * |
| Embryo size (mm^2^) | 4.04  (± 0.73) | 4.17  (± 0.91) | 4.67  (± 1.31) * | 4.71  (± 1.15) | 4.83  (± 0.95) † | 4.80  (± 1.23) | 4.35  (± 1.49) | 5.71  (± 1.00) * | 3.75  (± 0.88) | 5.12  (± 1.20) | 4.10  (± 0.64) ~~†~~ |
| Perisperm size (mm^2^) | 1.38  (± 0.57) | 1.40  (± 0.61) | 1.64  (± 0.58) * | 1.88  (± 0.95) | 1.59  (± 0.49) ^NS^ | 1.72  (± 0.57) | 1.56  (± 0.67) | 2.00  (± 0.57) * | 1.22  (± 0.44) | 1.92  (± 0.63) | 1.51  (± 0.41) ^NS^ |

Different genotypes specified in parentheses. * Best parent heterosis (*P* ≤ 0.05), † Mid-parent heterosis (*P* ≤ 0.05), ^NS^ Not significantly different (*P >* 0.05), ~~†~~ Below mid-parent value (*P* ≤ 0.05)

### Table S4. Agronomic and root quality traits of F1 triploid hybrids and their parent lines. Data are mean of four replicates (± SE).

|  | ♀ | ♂ | F1  3x hybrid (EAA) | ♂ | F1  3x hybrid (EBB) | ♀ | ♂ | F1  3x hybrid (FCC) | ♀ | ♂ | F1  3x hybrid (GDD) |
| --- | --- | --- | --- | --- | --- | --- | --- | --- | --- | --- | --- |
|  |  |  |  |  |  |  |  |  |  |  |  |
| Agronomic Traits | 2x  (EE) | 4x (AAAA) |  | 4x (BBBB) |  | 2x  (FF) | 4x (CCCC) |  | 2x  (GG) | 4x (DDDD) |  |
| Harvest plant density ^1^ | 46.5  (± 1.71) | 19.5  (± 3.20) | 43.75  (± 1.09) † | 18.5  (± 4.72) | 44.5  (± 2.06) † | 41.75  (± 2.50) | 44.75  (± 1.49) | 42.5  (± 1.44) ^NS^ | 43.25  (± 1.60) | 44.75  (± 0.25) | 44.5  (± 1.19) ^NS^ |
| Total yield (T/ha) | 121.79  (± 13.46) | 42.84  (± 10.39) | 142.68  (± 11.77) † | 33.68  (± 4.74) | 135.47  (± 11.83) † | 145.56  (± 11.32) | 144.98  (± 13.08) | 150.07  (± 7.02) ^NS^ | 128.10  (± 12.72) | 114.55  (± 3.87) | 160.37  (± 9.91) * |
| Above-ground biomass (g) ^2^ | 515.2  (± 63.79) | 463.63  (± 58.42) | 684.6  (± 7.79) * | 352.13  (± 28.90) | 608.8  (± 67.97) † | 935.6  (± 18.22) | 629.33  (± 52.42) | 779.4  (± 47.07) ~~*~~ | 795.4  (± 108.98) | 532.83  (± 19.65) | 764.48  (± 68.07) ^NS^ |
| Root length (cm) | 17.25  (± 0.58) | 15.85  (± 0.95) | 18.88  (± 0.60) * | 14.73  (± 0.42) | 17.91  (± 0.61) † | 17.97  (± 0.19) | 19.34  (± 0.16) | 18.15  (± 0.56) ~~*~~ | 16.67  (± 0.35) | 16.51  (± 0.57) | 17.64  (± 0.52) ^NS^ |
| Root circumference (cm) | 32.04  (± 1.36) | 29.98  (± 1.48) | 37.32  (± 0.65) * | 29.52  (± 1.75) | 35.73  (± 1.24) * | 34.33  (± 0.96) | 33.71  (± 1.05) | 34.94  (± 0.91) ^NS^ | 33.35  (± 2.11) | 29.29  (± 0.53) | 34.54  (± 1.05) ^NS^ |
| Root yield (T/ha) | 71.44  (± 7.13) | 24.87  (± 6.19) | 85.81  (± 7.92) † | 20.24  (± 3.26) | 82.03  (± 6.90) † | 70.87  (± 7.18) | 87.92  (± 8.72) | 84.46  (± 4.97) ^NS^ | 64.90  (± 6.54) | 64.31  (± 2.82) | 87.51  (± 5.78) * |
| Root Quality Traits |  |  |  |  |  |  |  |  |  |  |  |
| Corrected sugar content (%) | 14.74  (± 0.40) | 12.84  (± 0.30) | 13.87  (± 0.09) ^NS^ | 12.87  (± 0.16) | 13.98  (± 0.24) ^NS^ | 15.40  (± 0.19) | 14.36  (± 0.33) | 14.77  (± 0.26) ^NS^ | 14.85  (± 0.31) | 14.71  (± 0.07) | 14.87  (± 0.39) ^NS^ |
| Standard molasses loss (%) | 1.85  (± 0.07) | 2.19  (± 0.07) | 1.86  (± 0.02) ^†^ | 1.93  (± 0.10) | 1.81  (± 0.06) ^NS^ | 1.73  (± 0.05) | 1.90  (± 0.09) | 1.77  (± 0.03) ^NS^ | 1.84  (± 0.06) | 1.68  (± 0.05) | 1.69  (± 0.06) ^NS^ |
| Corrected sugar yield (T/ha) | 10.44  (± 0.78) | 3.14  (± 0.74) | 11.89  (± 1.04) † | 2.59  (± 0.40) | 11.60  (± 1.23) † | 10.62  (± 1.47) | 12.53  (± 0.92) | 12.44  (± 0.55) ^NS^ | 9.57  (± 0.85) | 9.46  (± 0.40) | 12.99  (± 0.81) * |

Different genotypes specified in parentheses. * Best parent heterosis (*P* ≤ 0.05), † Mid-parent heterosis (*P* ≤ 0.05), ^NS^ Not significantly different (*P >* 0.05), ~~*~~ Below best parent value (*P* ≤ 0.05), ~~†~~ Below mid-parent value (*P* ≤ 0.05)
