# Supplementary material for: Hybridity has a greater effect than paternal genome dosage on heterosis in sugar beet (Beta vulgaris)
Source: BMC Plant Biol. 2018 Jun 15;18:120. doi: 10.1186/s12870-018-1338-x (PMC6003118; doi:10.1186/s12870-018-1338-x)
Supplement: Supplementary file 4 — Fig. S4.1. Top and bottom view of representative alive and dead sugar beet seeds. Fig. S4.2. Flow cytometry analysis of nuclei from leaves confirms ploidy level of each line. Fig. S4.3. Germination test shows F1 diploid hybrids of sugar beet display heterosis effects on seed germination. Germination test shows there is a heterosis effect on germination in F1 3× hybrid (EAA) but not F1 3× hybrid (EBB). Fig. S4.4. F1 triploid hybrids with heterozygous tetraploid male parents display mid-parent heterosis in relation to early germination. (DOCX 7935 kb). [file 12870_2018_1338_MOESM4_ESM.docx]

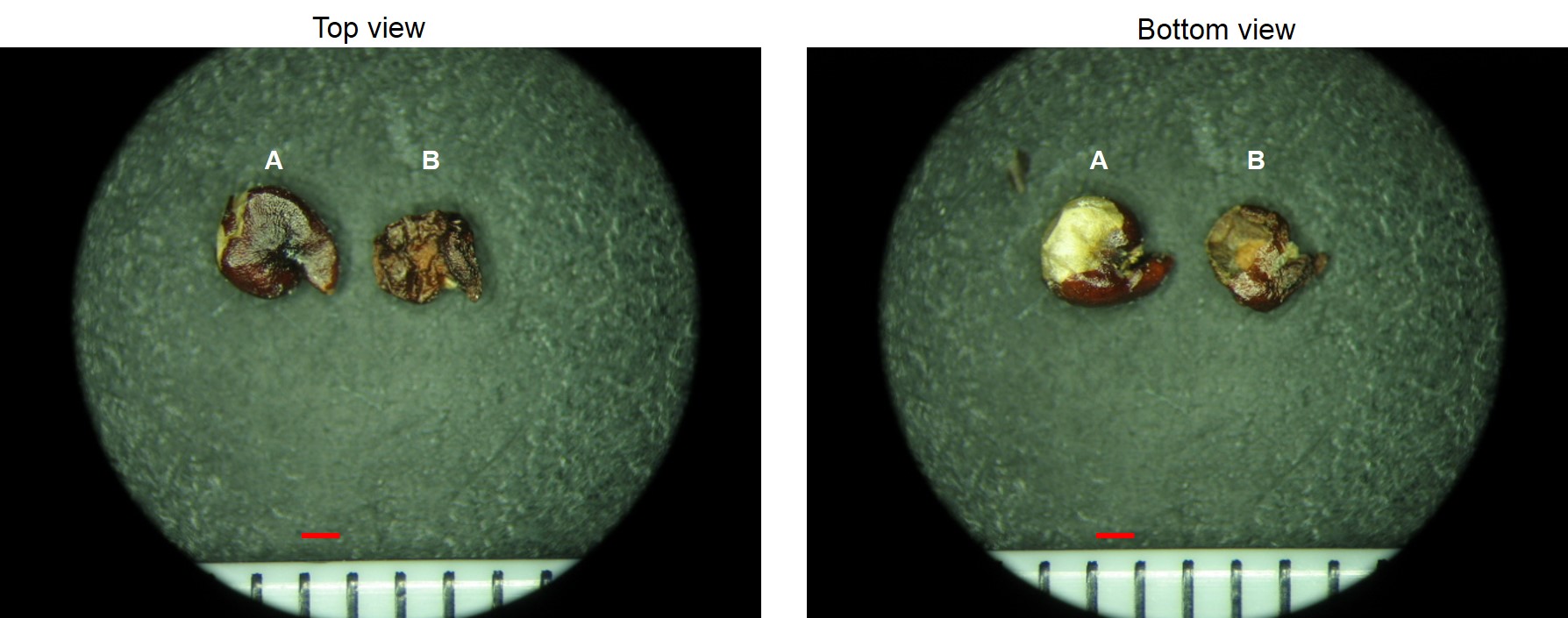


**Figure S4.1** Top and bottom view of representative **A** alive seed and **B** dead seed. Red line is scale of 1.00mm.

**Figure S4.2 Flow cytometry analysis of nuclei from leaves confirms ploidy level of each line**


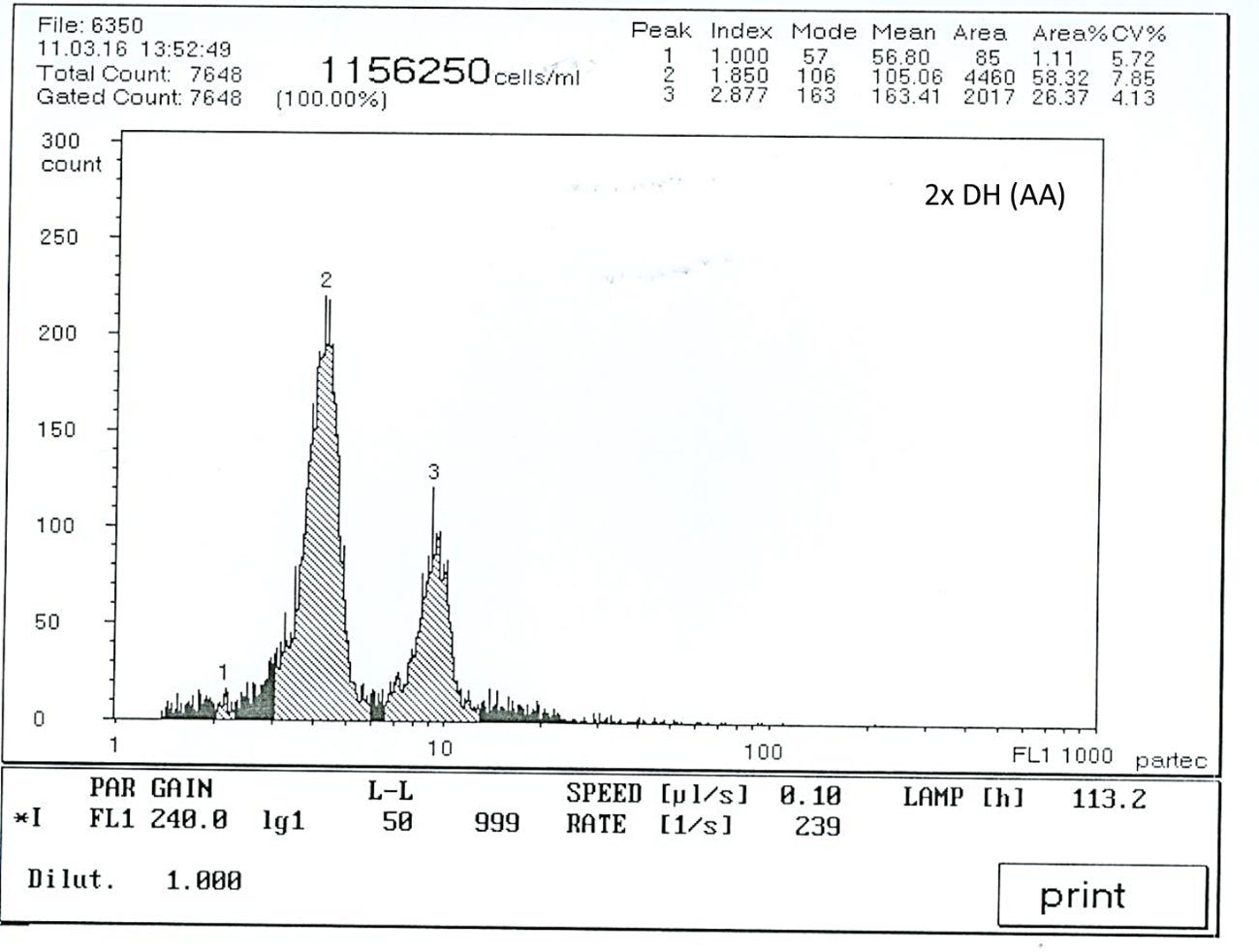


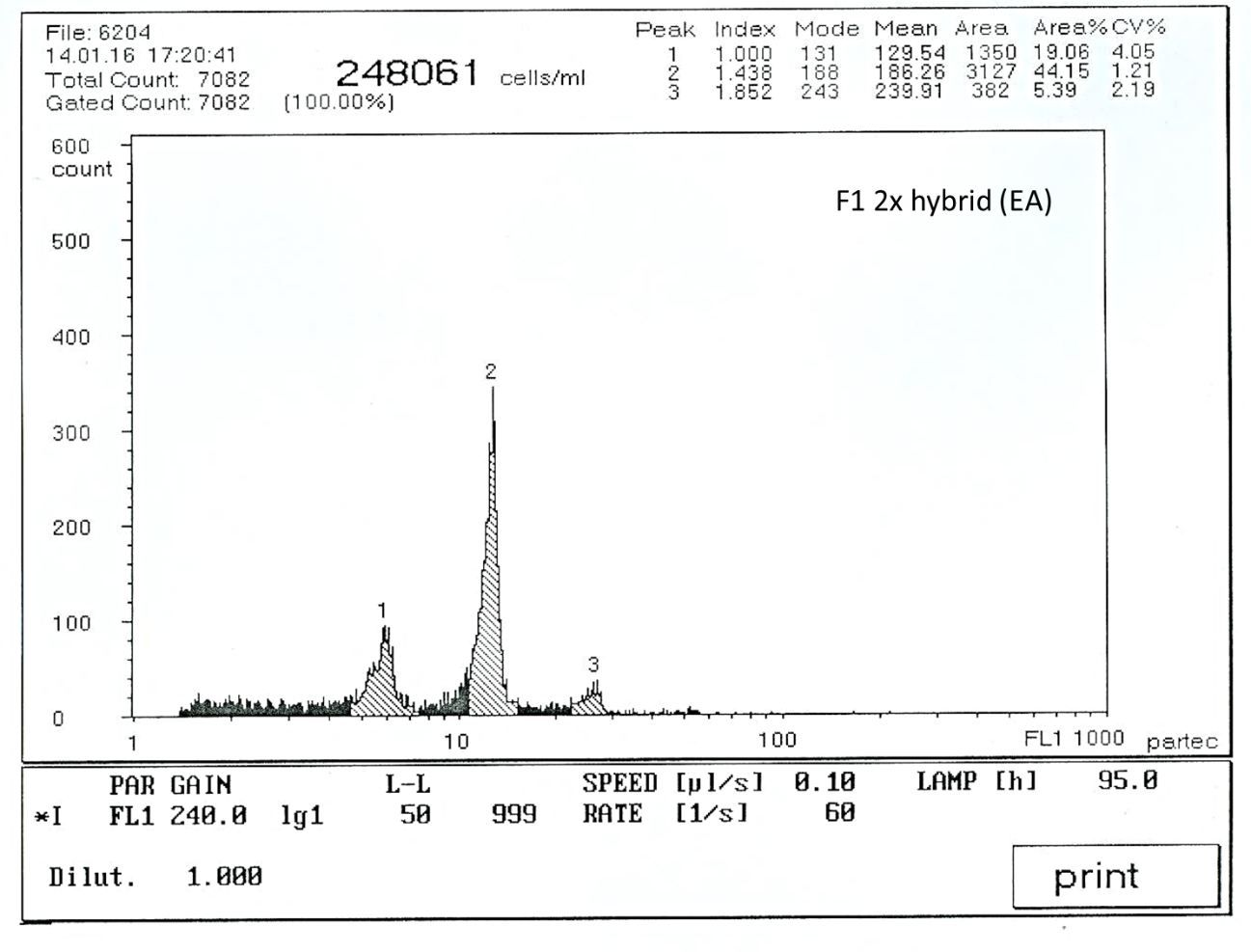


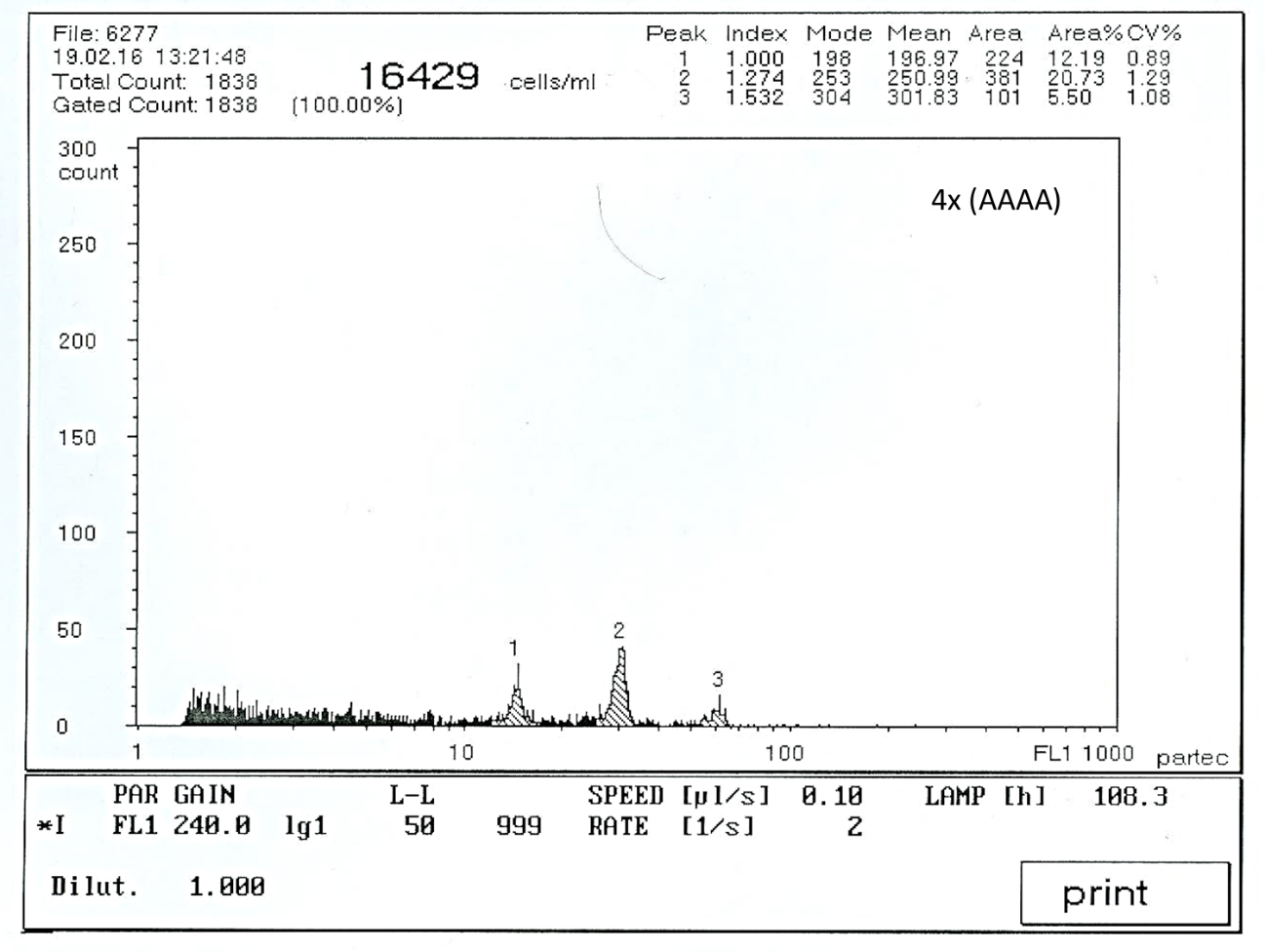


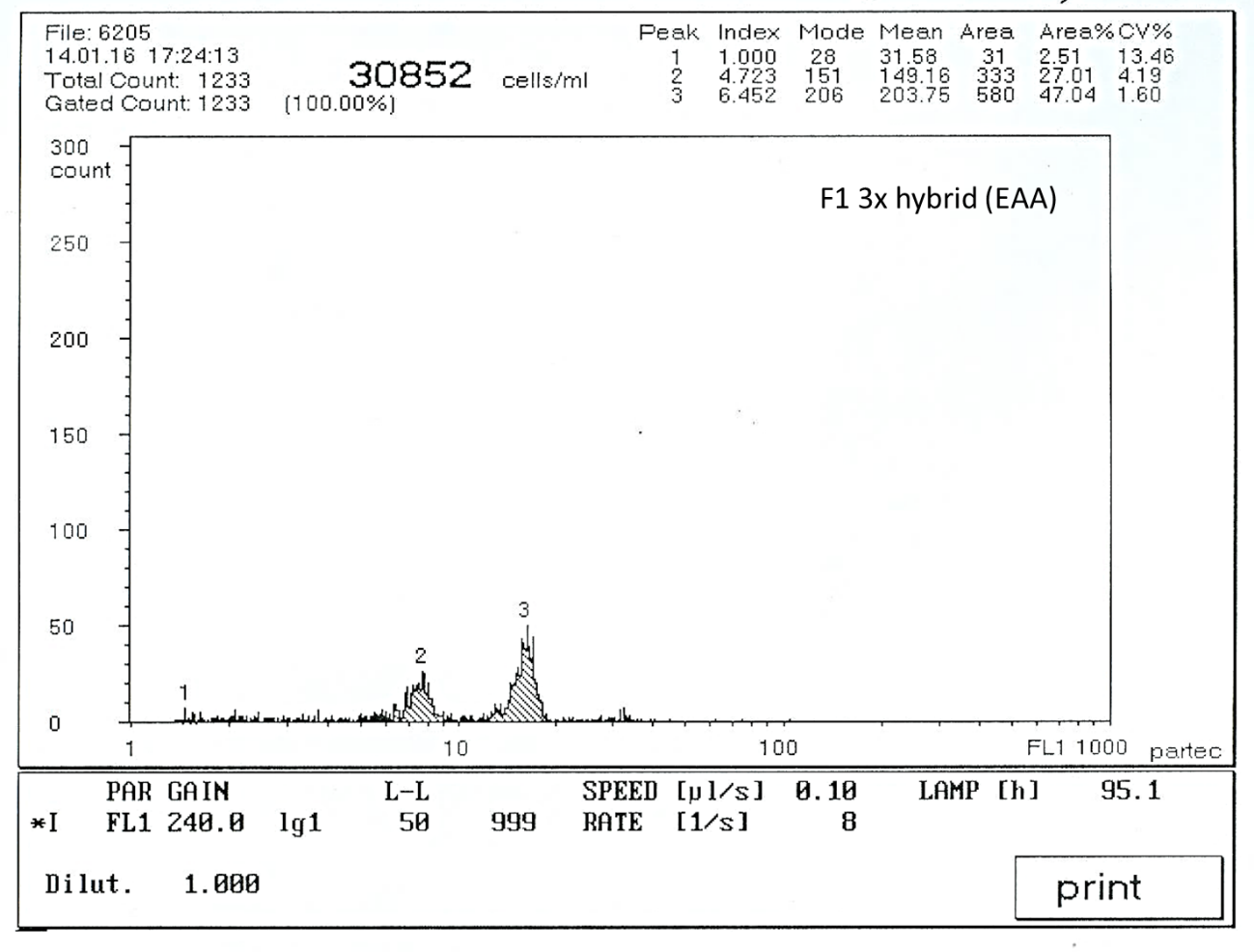


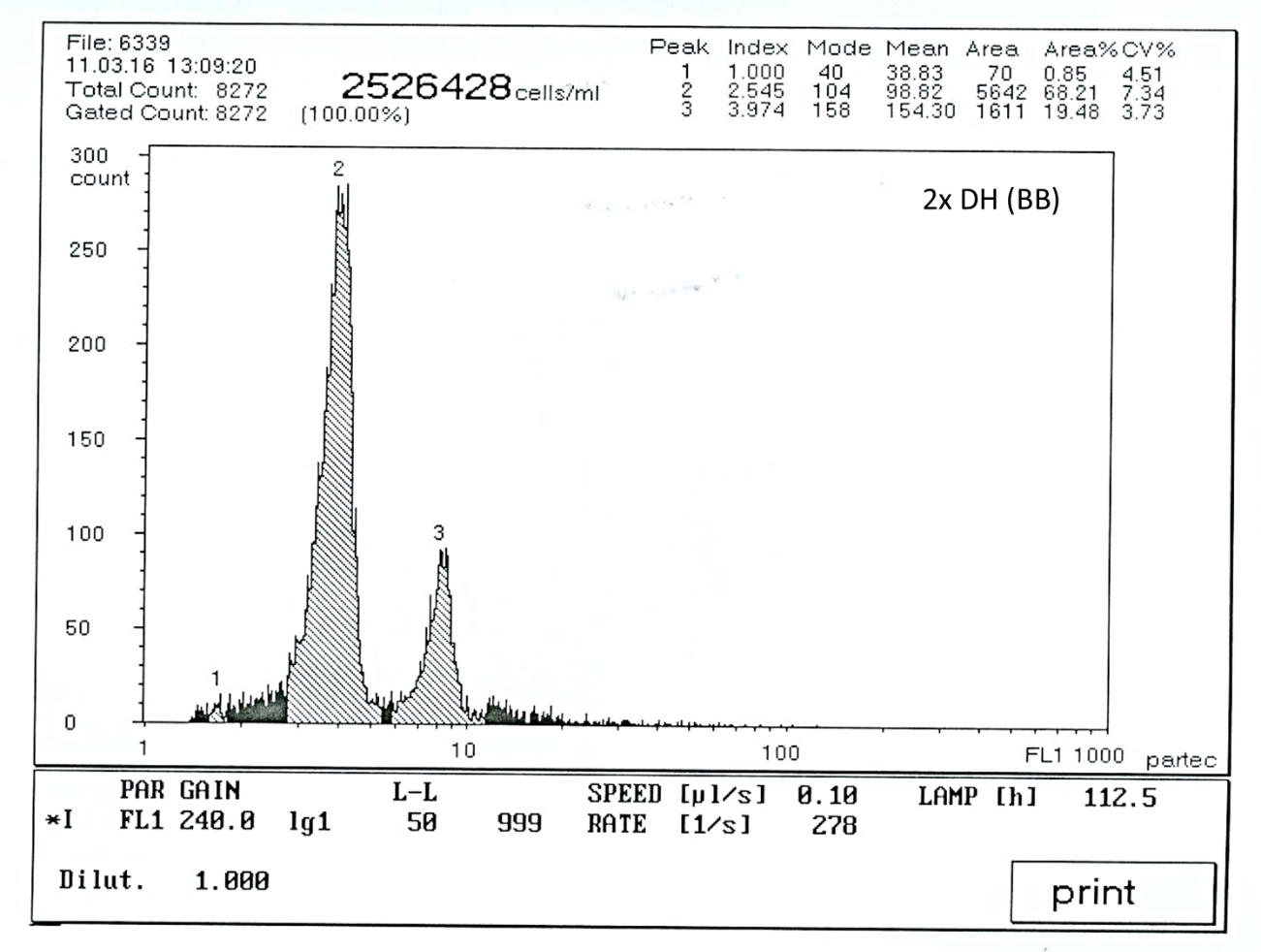


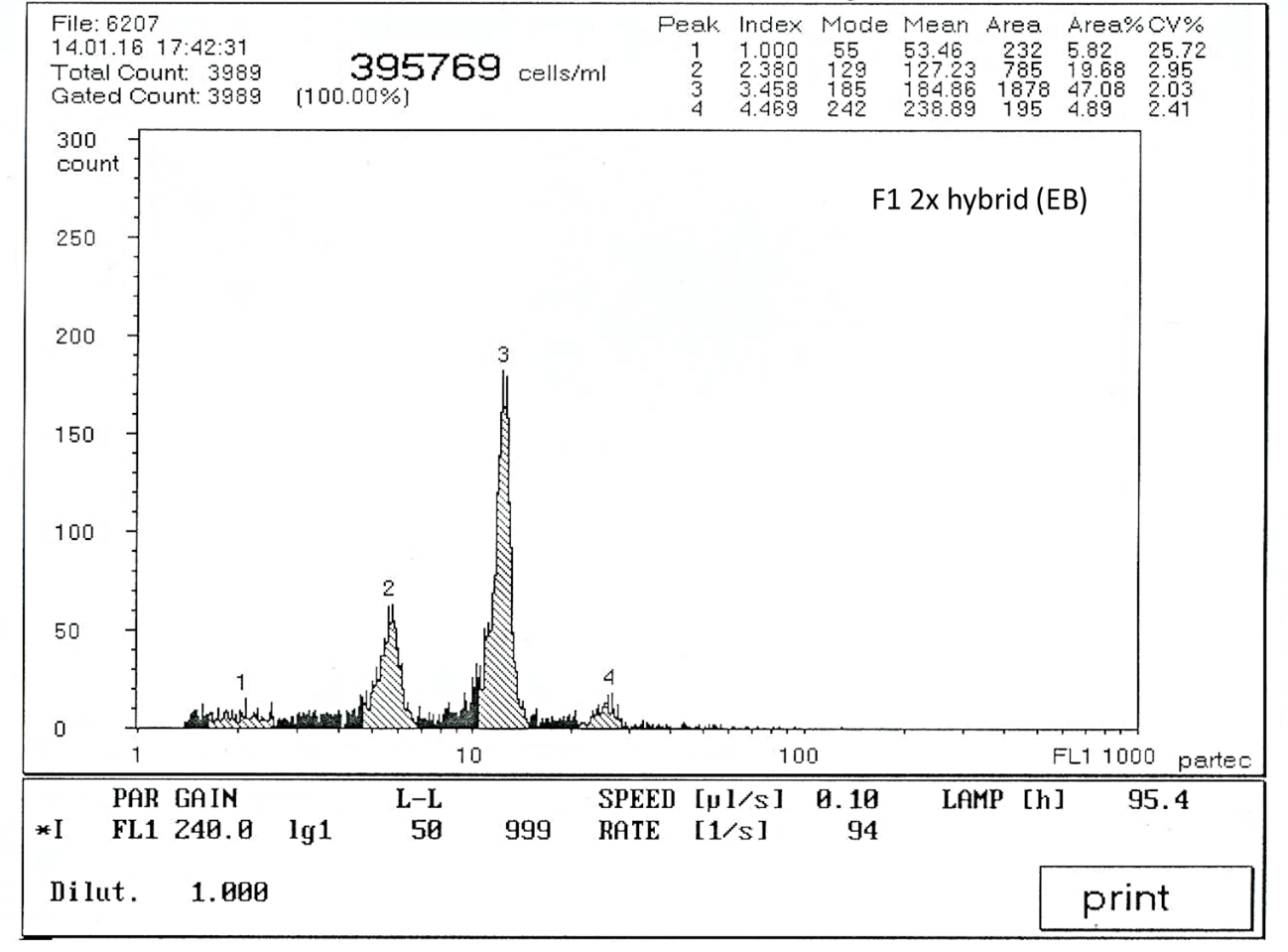


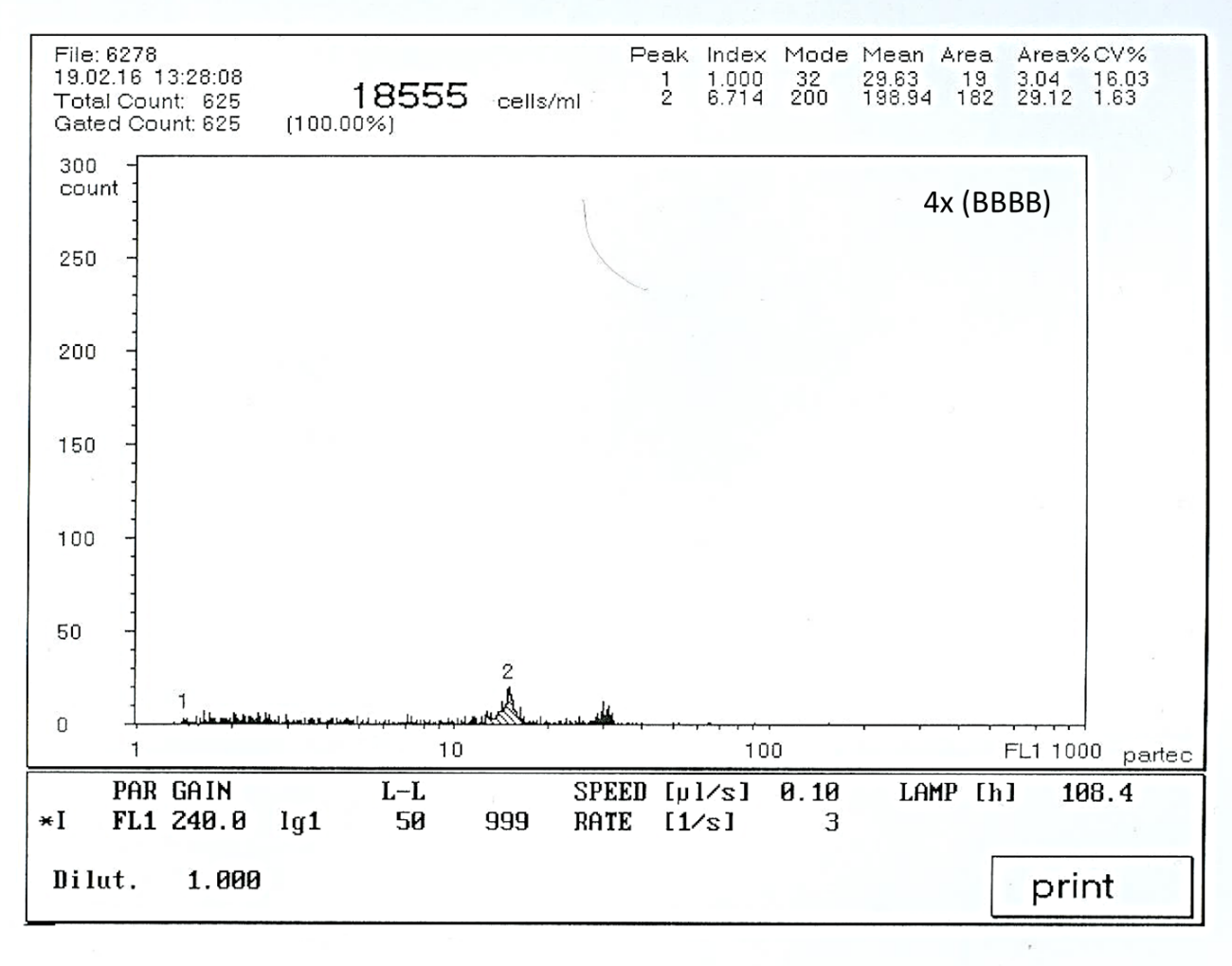


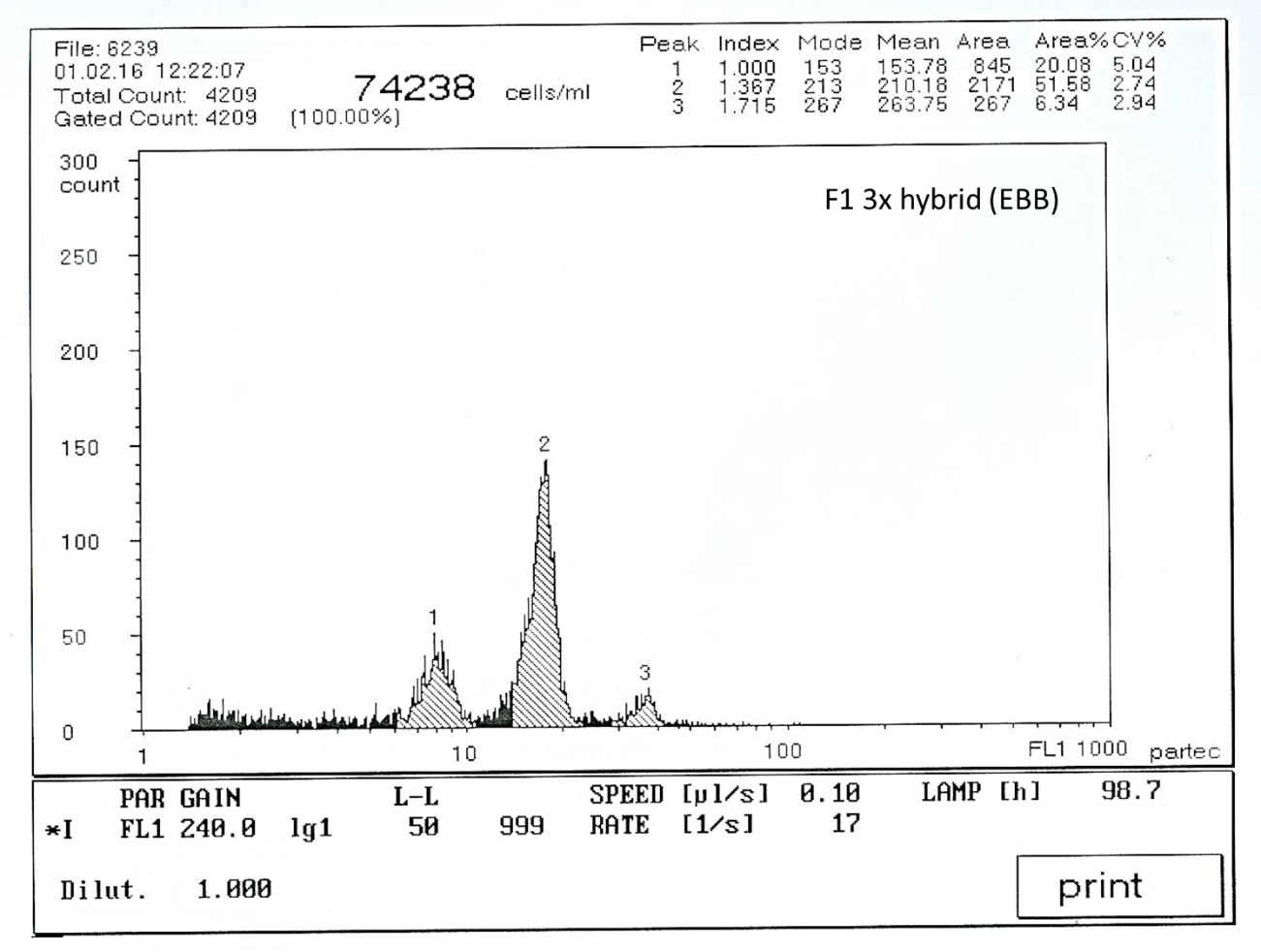


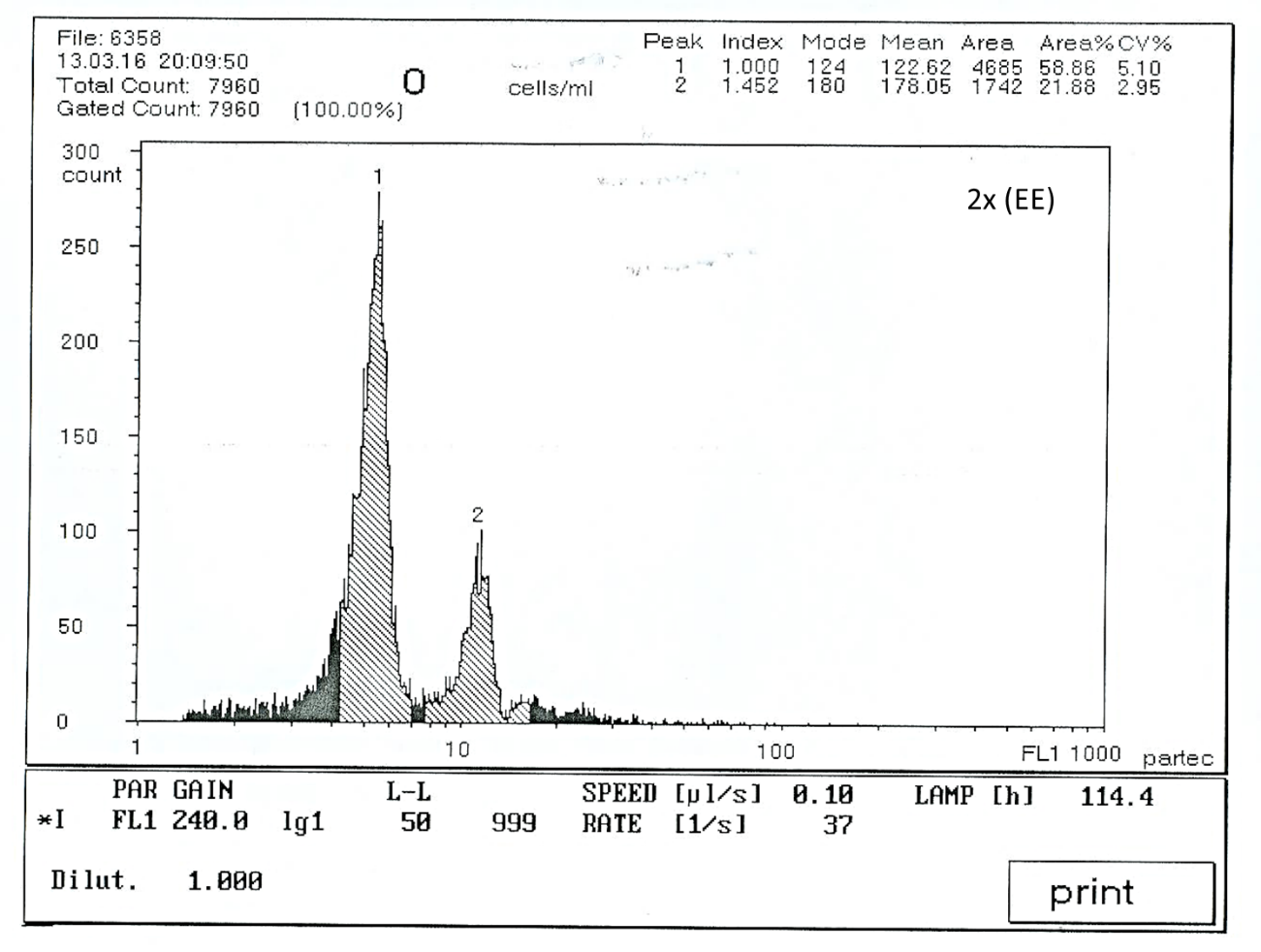


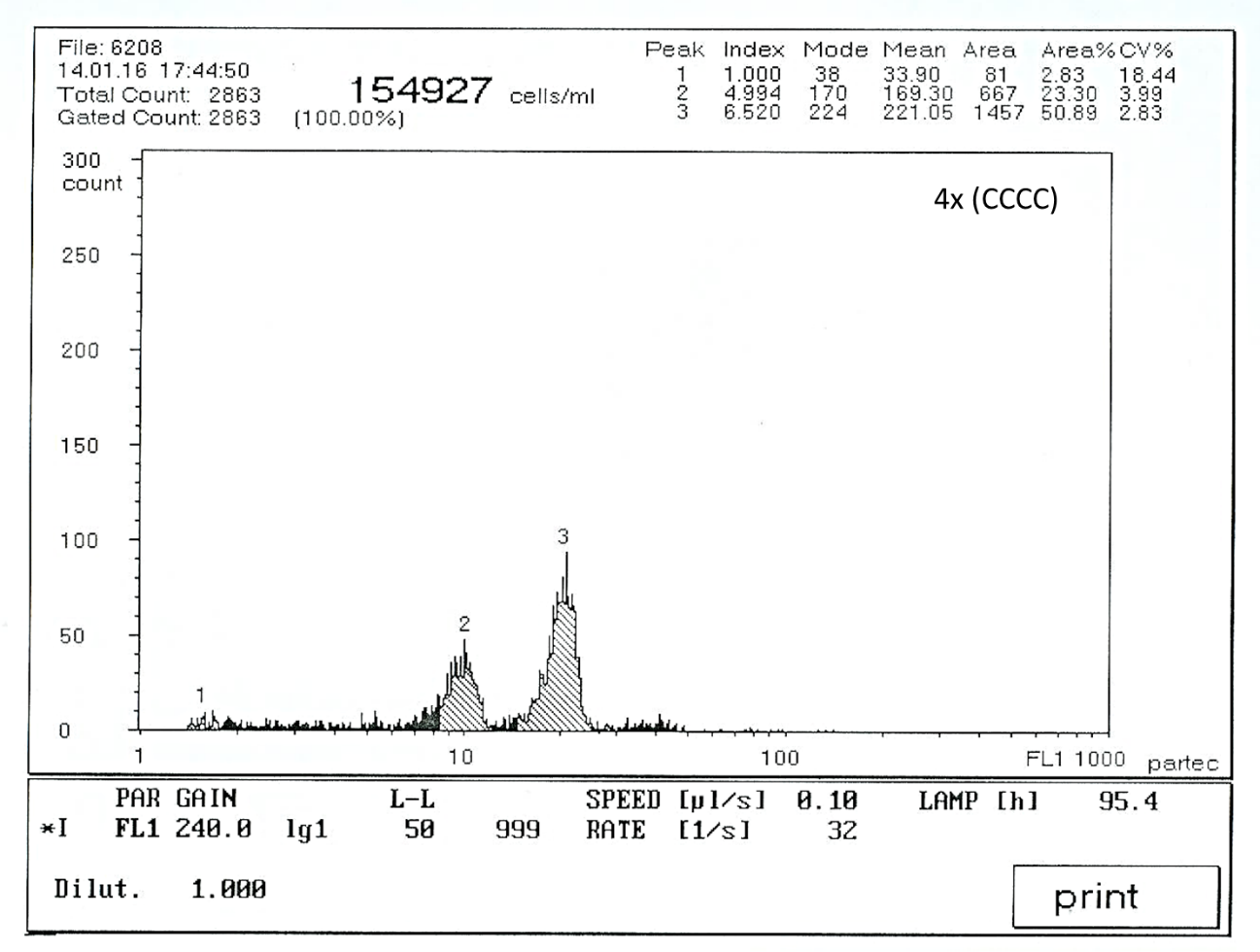


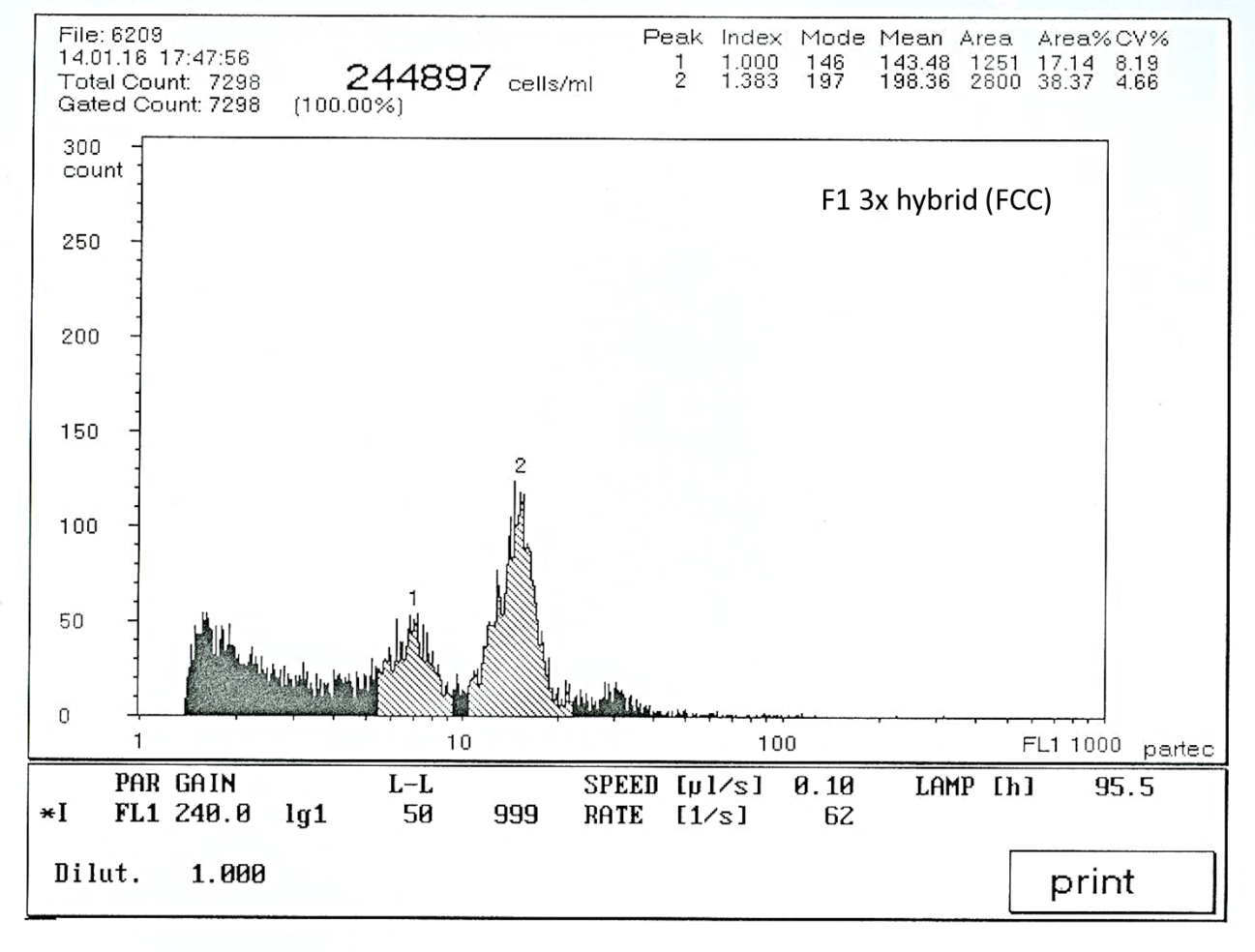


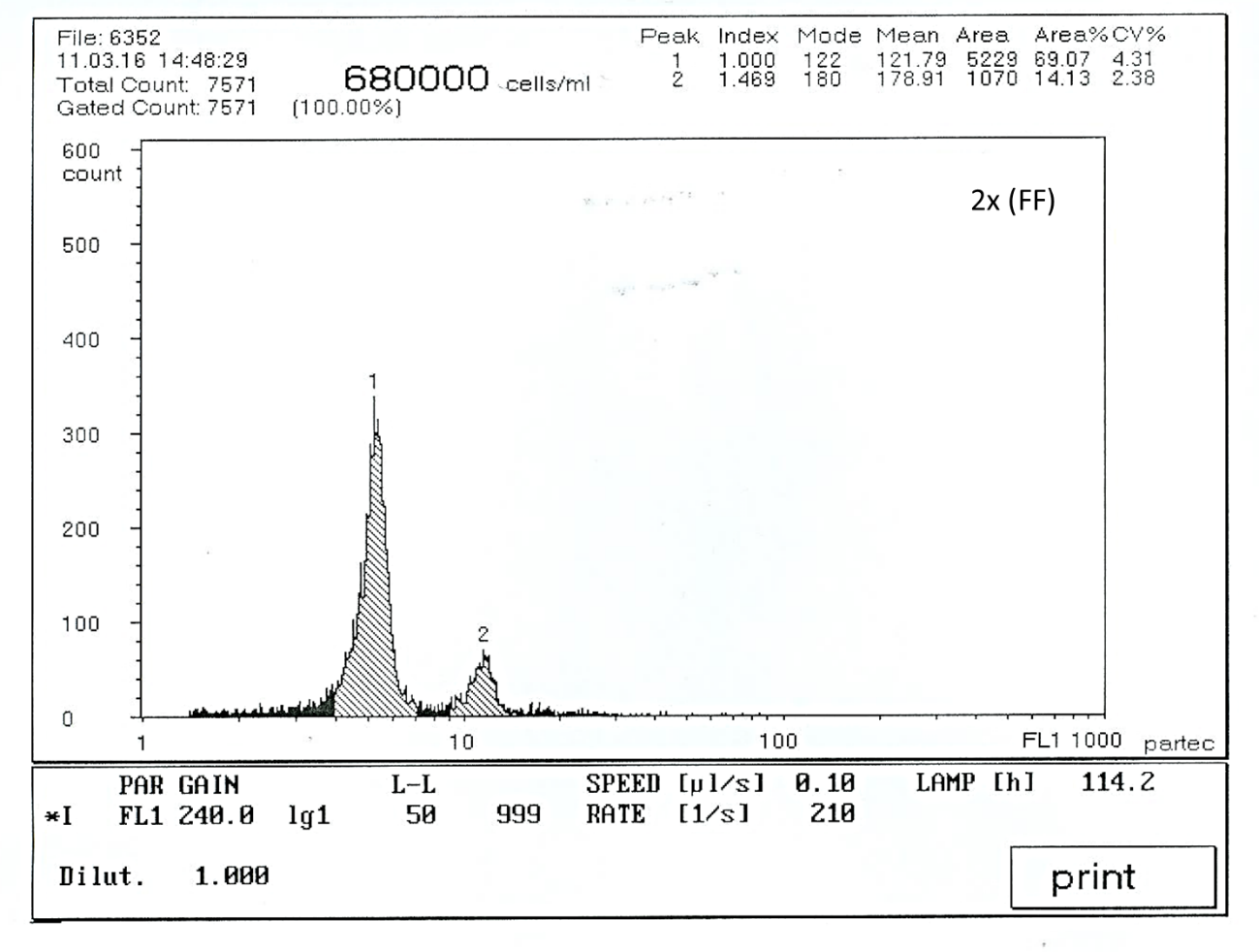


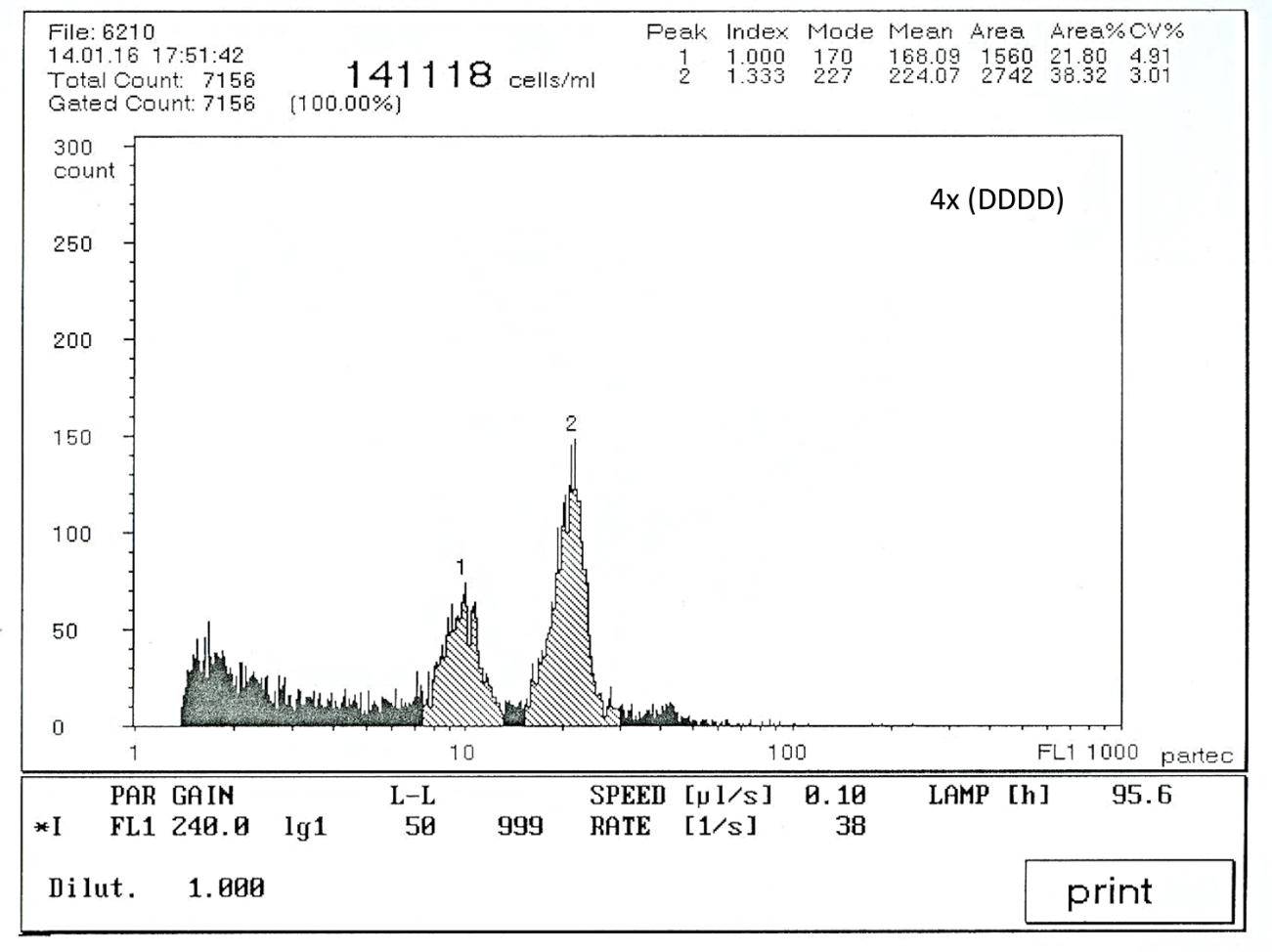


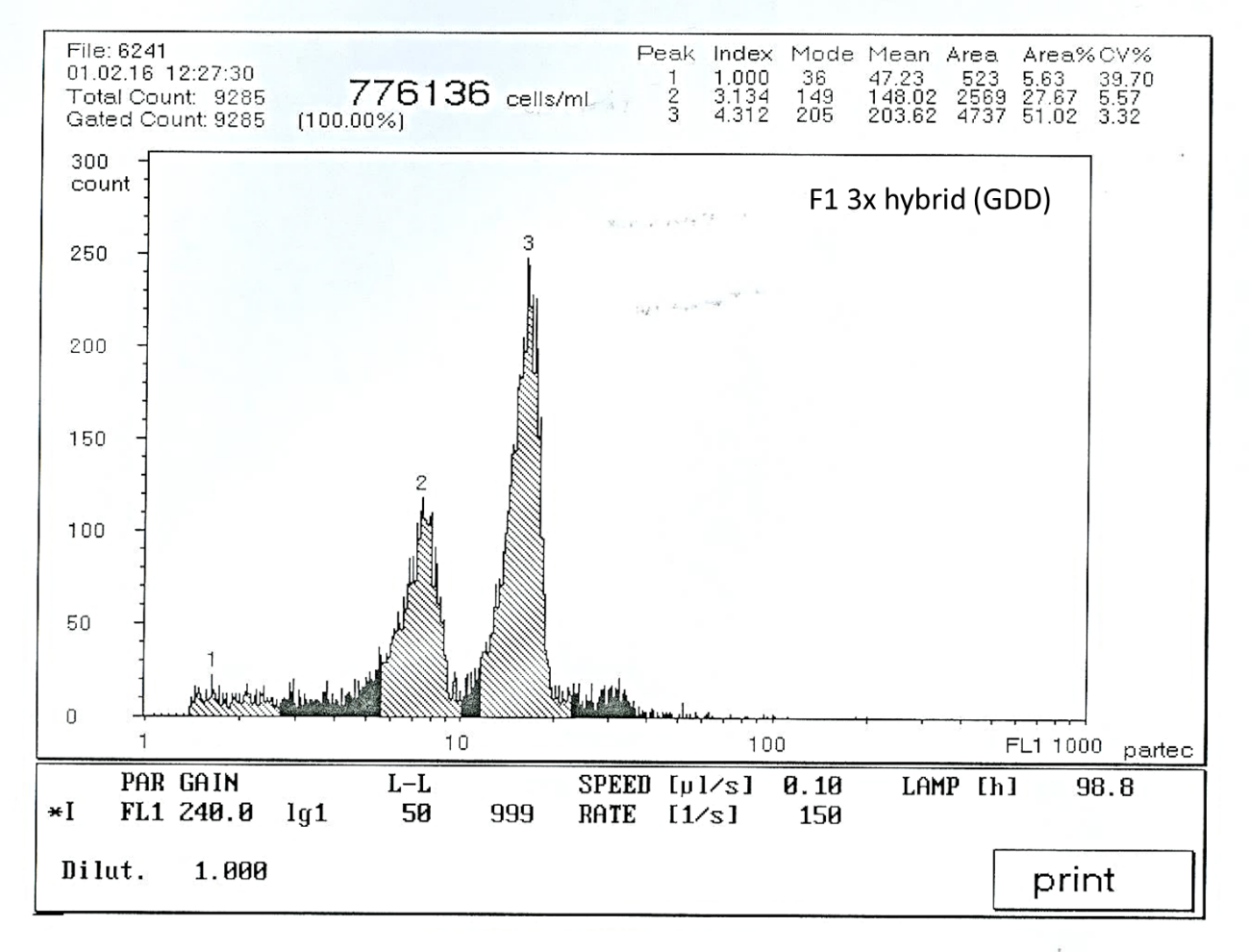


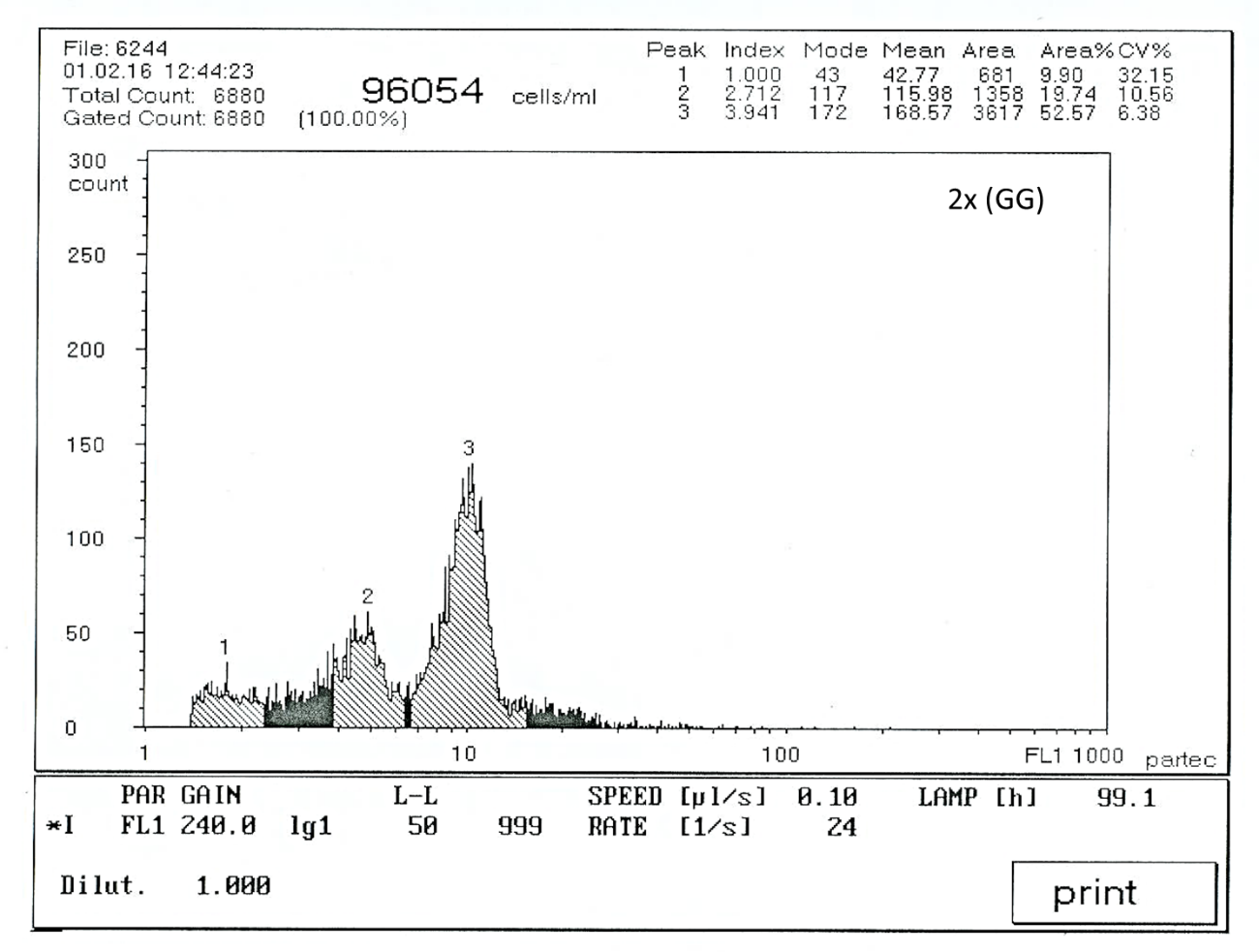


**F1 diploid hybrids of sugar beet can display heterosis effects on seed germination**

To determine whether F1 hybrid seeds at the diploid level displayed heterosis effects on seed germination, seeds of both parents and F1 hybrids were comparatively germinated using standard procedures. The F1 2x hybrid (EA) displayed heterosis for germination (*P* ≤ 0.05) on day 4 (92%) and day 14 (97%), while the F1 2x hybrid (EB) did not display any significant heterosis effects on germination (*P* > 0.05) at both day 4 and 14.

**There is a heterosis effect on germination in F1 3x hybrid (EAA) but not F1 3x hybrid (EBB)**

F1 3x hybrid (EAA) displays heterosis for germination (*P* ≤ 0.05) on day 4 (85%) like its equivalent F1 2x hybrid (EA), but at day 14 is not significantly different (*P* = 0.16) from the mid-parent value. There is no heterosis effect on germination in F1 3x hybrid (EBB) which is also seen in its equivalent F1 2x hybrid (EB) (Figure **3**). While 4x (AAAA) and (BBBB) achieve ~50% germination at day 14, the seedlings of these genotypes were small and weak.

**
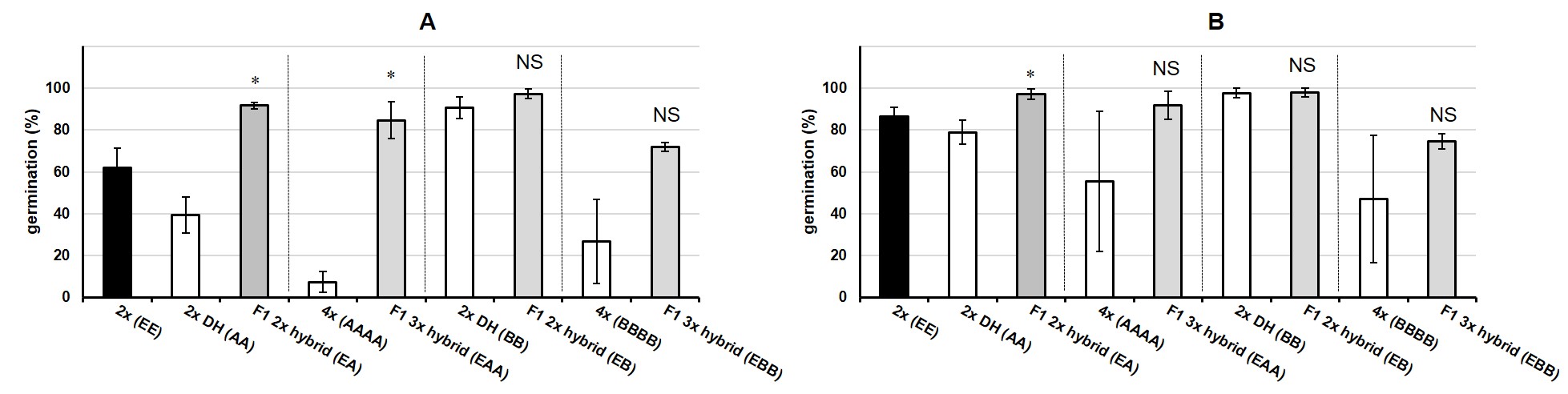
Figure S4.3 Germination percentage of F1 diploid and triploid hybrids and their parent lines.** Data are mean of three replicates (± SD). **A** Day 4, **B** day 14. Different genotypes specified in parentheses. DH = double haploid. * Best parent heterosis (P ≤ 0.05), NS Not significantly different (P > 0.05).

**F1 triploid hybrids with heterozygous tetraploid male parents display mid-parent heterosis in relation to early germination**

F1 3x hybrid (FCC) and (GDD) reach 76% and 91% germination respectively at day 4. Both these percentages are greater than their mid-parent values (*P* ≤ 0.05). Both heterozygous male parents, 4x (CCCC) and (DDDD), achieve almost maximum germination at day 4 (A). At day 14, there is no significant difference between F1 3x hybrids and their mid-parent values (*P* > 0.05) and all heterozygous parents have 86-96% germination (B).


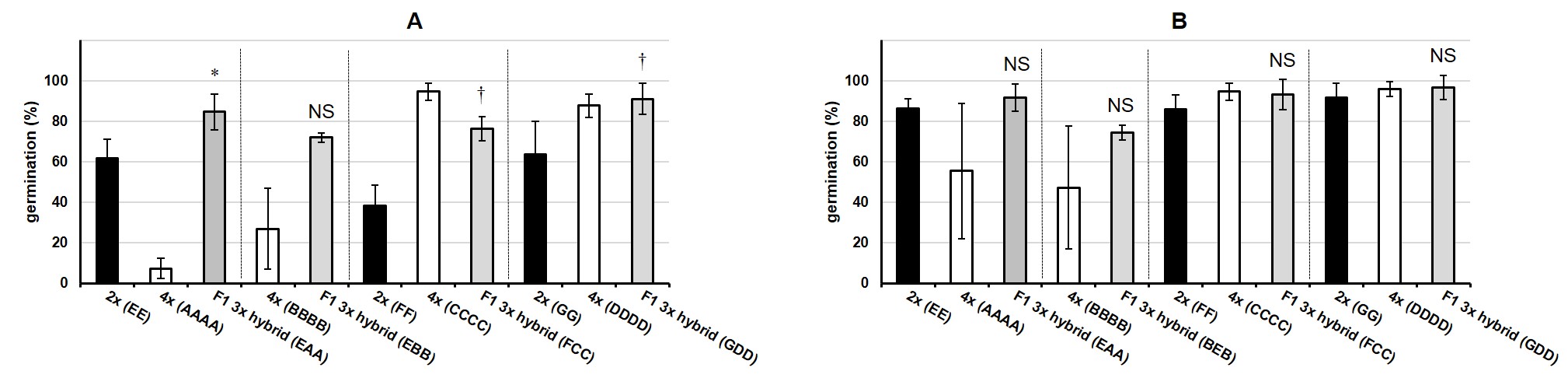


**Figure S4.4 Germination percentage of F1 triploid hybrids and their parent lines.** Data are mean of three replicates (± SD). **A** Day 4, **B** day 14. Different genotypes specified in parentheses. * Best parent heterosis (P ≤ 0.05), † Mid-parent heterosis (P ≤ 0.05), NS Not significantly different (P > 0.05).
